# Supplementary material for: Targeting long non-coding RNA MALAT1 preserves endothelial cell integrity and protects against kidney fibrosis
Source: Mol Ther Nucleic Acids. 2025 Aug 19;36(3):102689. doi: 10.1016/j.omtn.2025.102689 (PMC12445227; doi:10.1016/j.omtn.2025.102689)
Supplement: Document S1. Figures S1–S13 [file mmc1.pdf]

OMTN, Volume *36*

Supplemental information

Targeting long non-coding RNA *MALAT1* preserves endothelial cell integrity and protects against kidney fibrosis

Qiao Zhao, Loïs A.K. van der Pluijm, Morgane Gourvest, Atefeh Lafzi, Daniel Peled, Whitney G. Rubin, Juliette A. de Klerk, Roderick C. Slieker, Leen M. 't Hart, Wendy Stam, Annemarie M. van Oeveren-Rietdijk, Jacques M.G.J. Duijs, Angela Koudijs, Joris I. Rotmans, Hilal Kazan, Anton Jan van Zonneveld, Coen van Solingen, and Roel Bijkerk

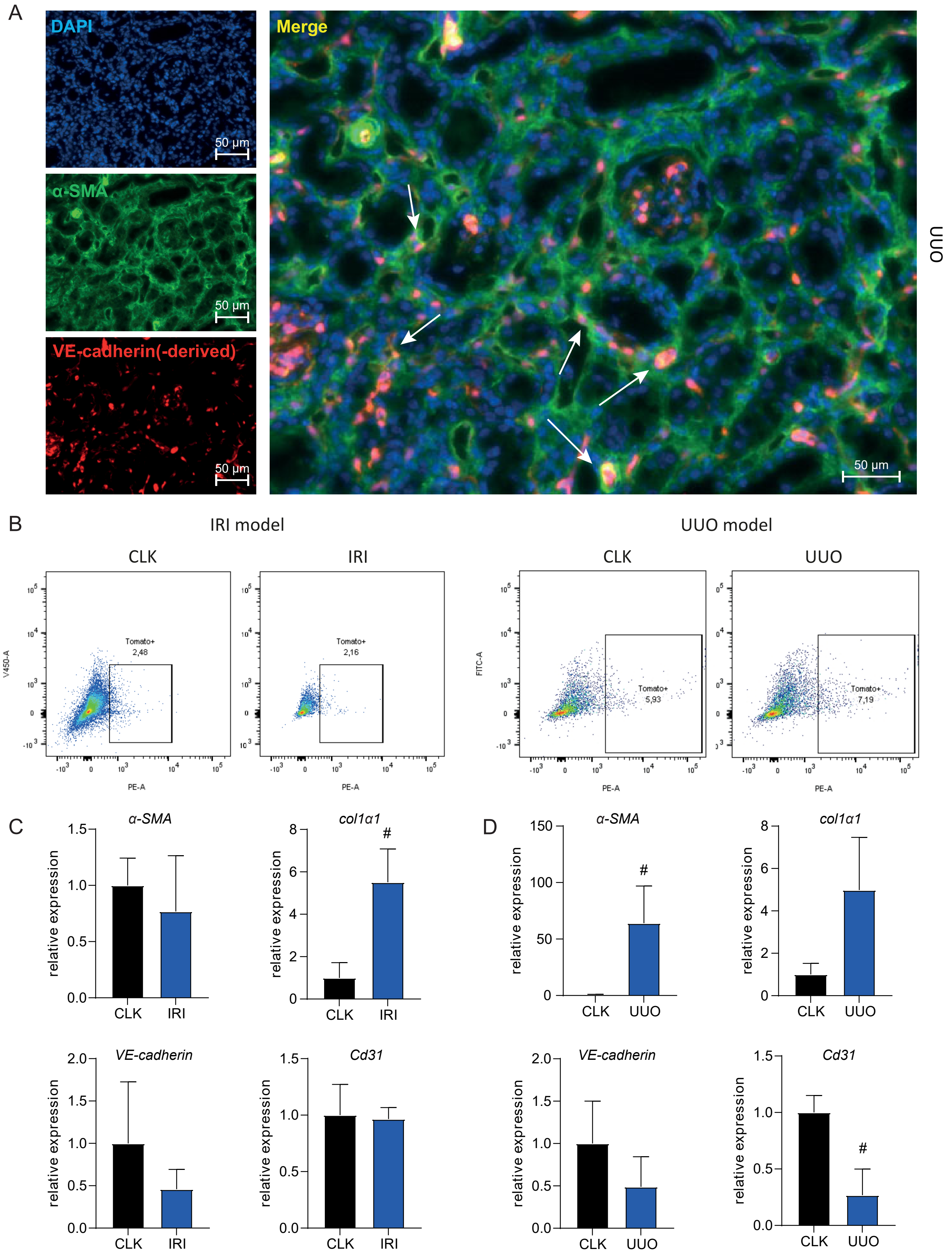

Figure S1. Endothelial-to-mesenchymal transition in murine kidney fibrosis. (A) representive image of co-localization (examples are indicated with arrows) of VE-cadherin-derived tomato positive cells and α-SMA stained positive cells in a UUO kidney. (B) Representative FACS plots for isolation of Tomato-positive VE-Cadherin-derived cells. (C-D) qPCR was performed for endothelial cell genes (*Cd31* and *Cdh5* (*VE-cadherin*)) and mesenchymal/ myofibroblast genes (*Acta2* (*α-SMA*) and *Col1α1*) on FACS sorted cells from VE-cadherin-tomato mice in the IRI model (C) and UUO model (D). $^{\#}$P<0.10.

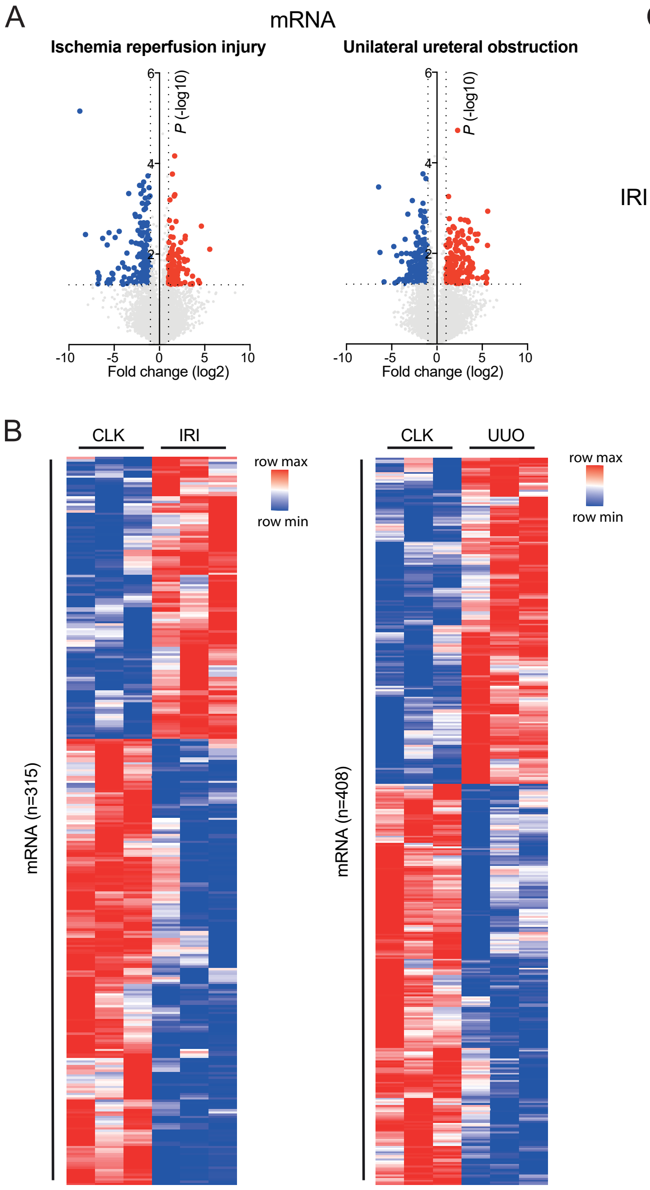

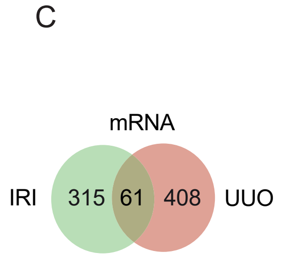

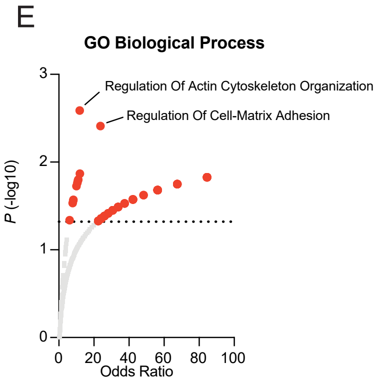

D

IRI

Pathway	p-value
STAT3 Pathway	1.30E-04
S100 Family Signaling Pathway	3.62E-04
Molecular Mechanisms of Cancer	4.03E-04
WNK Renal Signaling Pathway	5,60E-04
Selenoamino acid metabolism	7.37E-04

UUO

Pathway	p-value
RhoA Signaling	2.32E-05
RHO GTPase cycle	4.66E-05
NCAM signaling for neurite out-growth	3.00E-04
S100 Family Signaling Pathway	1.30E-03
Triacylglycerol Degradation	1.58E-03

Figure S2. Differential mRNA expression in endothial (-derived) cells in IRI and UUO. (A) Volcano Plots are visualizing differential mRNA expression between indicated conditions. The blue and red dots correspond to mRNAs with $P<0.05$ and $-1<logFC>1$ that are down or up regulated, respectively. (B) Hierarchical clustering shows a distinguishable mRNA expression pattern in VE-cadherin derived cells in IRI and UUO compared to healthy contralateral kidneys (CLK) (n=3 per condition). (C) VENN diagram showing total number of differentially expressed genes per model as well as overlapping genes. (D) Ingenuity Pathway analyses indicates predicted enriched pathways. (E) GO analysis of biological pathways among the 61 overlapping genes between IRI and UUO are involved in actin cytoskeleton regulation and cell-matrix iadhesion.

A

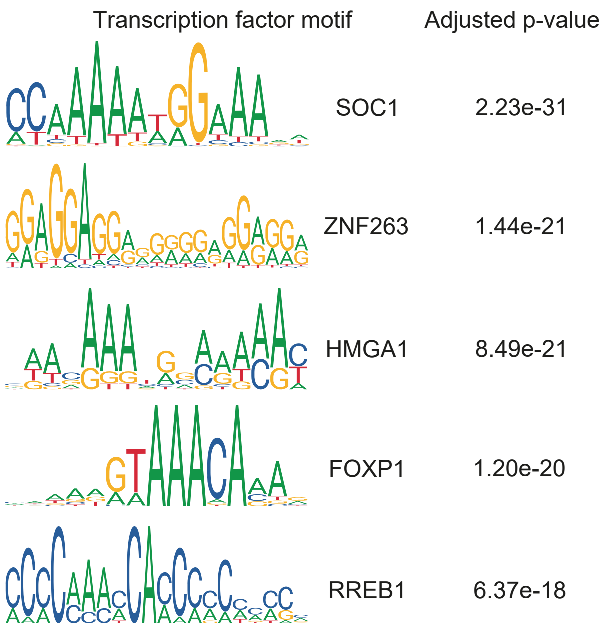

B

-821 ......TGGGAGAAAGAGAAAAACATCCTTT...... -796

Green - HMGA1 motif JASPAR

-1691 ......GAAAAATAAATAAGGAAGAAAGAAAA...... -1665

Red - HMGA1 motif (Reeves, 2000)

-249 ......TTGTGACCAGAAATTTTAAACCTATTT...... -222

Blue - HMGA1 motif (Manabe, 2009)

C

Upstream Regulator	Predicted Activation State	Activation z-score
HMGA1	Inhibited	-2.000
LPS	Activated	2.369
OSM	Activated	2.164
CST5	Activated	2.000

D

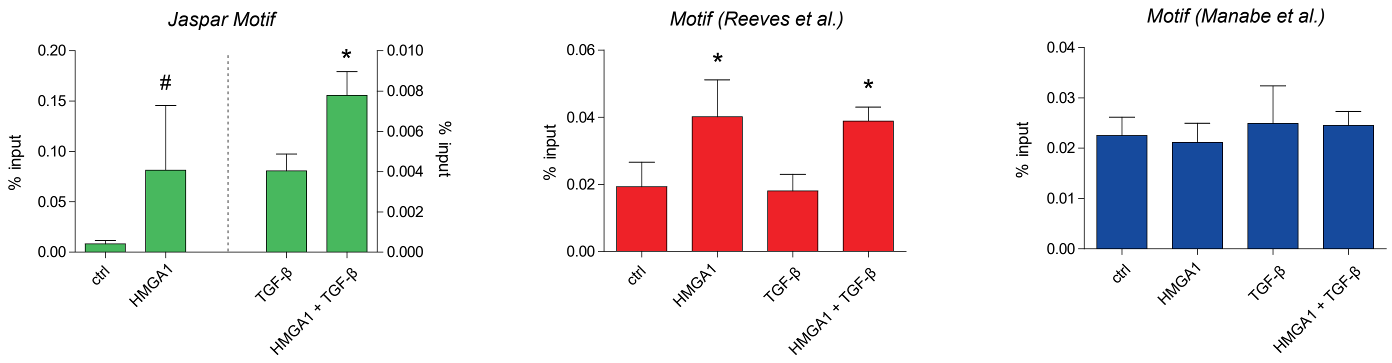

Figure S3. (A) Transcription factor motif enrichment analysis (JASPAR Core 2016) indicates enriched transcription factor motifs in promoter regions of differentially expressed lncRNAs. Top 5 (p-value) is depicted. (B) Within the promoter region of *Malat1* (defined as <2000 bp upstream of the start site) we found 3 potential HMGA1 binding motifs. In addition to the identified JASPAR motif for HMGA1, two additional potential binding motifs for HMGA1 were identfied from literature (Reeves, 2000, Environ Health Perspect & Manabe *et al.*, 2009, PLoS One). (C) Pathway analysis on differential mRNA profiles (Supplementary Figure 2) predicts HMGA1 to be a negatively regulated upstream regulator of differentially expressed genes in ECs in UUO. (D) PCR for *Malat1* promoter regions following HMGA1 ChIP, with IgG as negative control. *P<0.05, #P<0.10

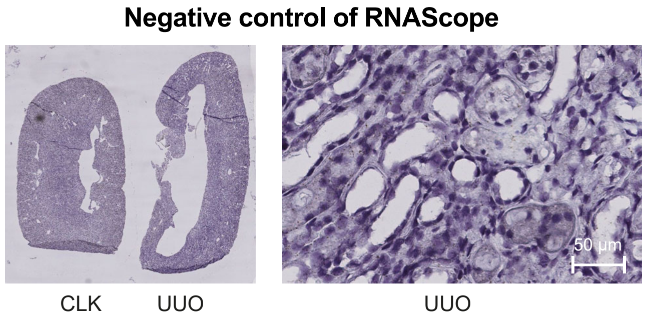

Figure S4. Negative control of RNAscope.

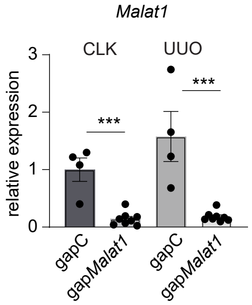

Figure S5. RT-qPCR analysis of *Malat1* in the kidney upon *Malat1* targeting GapmeR treatment (gap*Malat1*), compared to control GapmeR (gapC) treated mice. ***P<0.001. CLK = healthy contralateral kidney, UUO = fibrotic kidney from unilateral ureteral obstruction model. gapC = control GapmeR, gap*Malat1* = *Malat1* GapmeR.

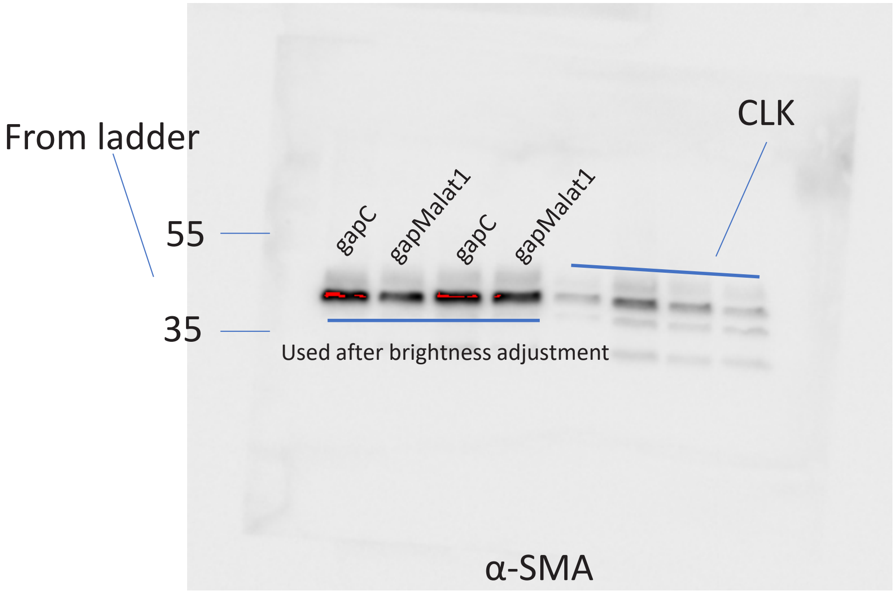

From ladder
55
35
gapC
gapMalat1
gapC
gapMalat1
CLK
Used after brightness adjustment
α-SMA

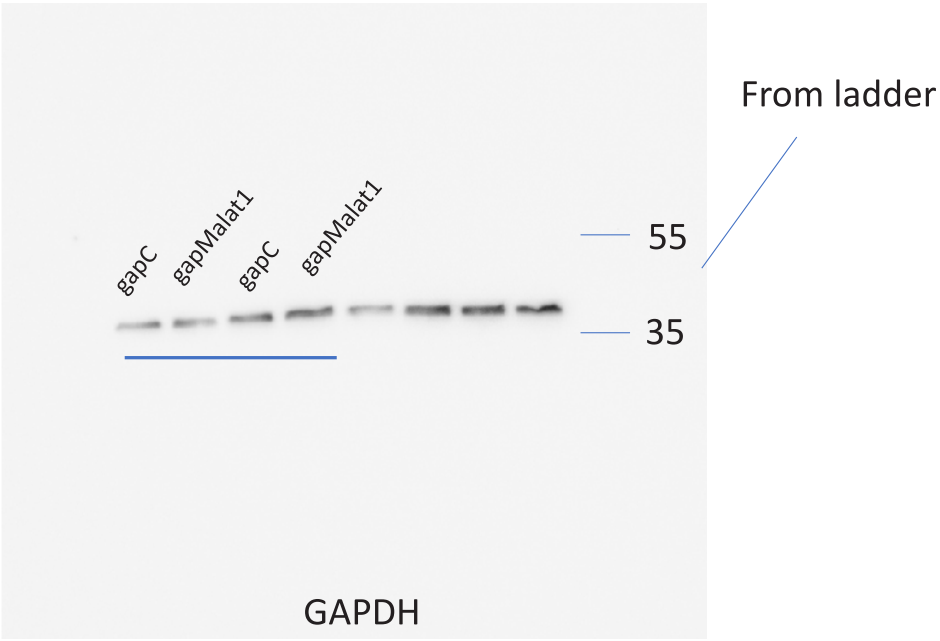

gapC
gapMalat1
gapC
gapMalat1
From ladder
55
35
GAPDH

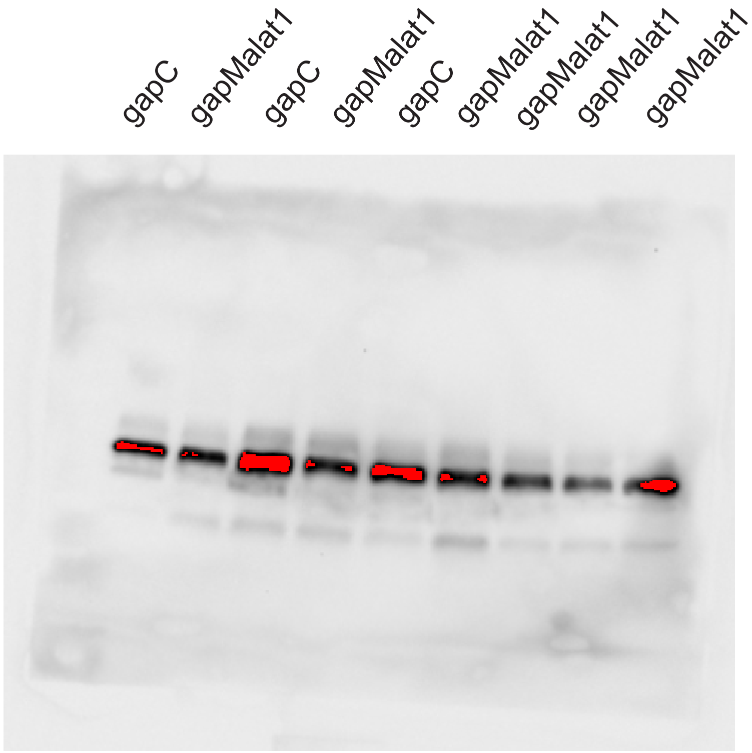

α-SMA

Additional samples

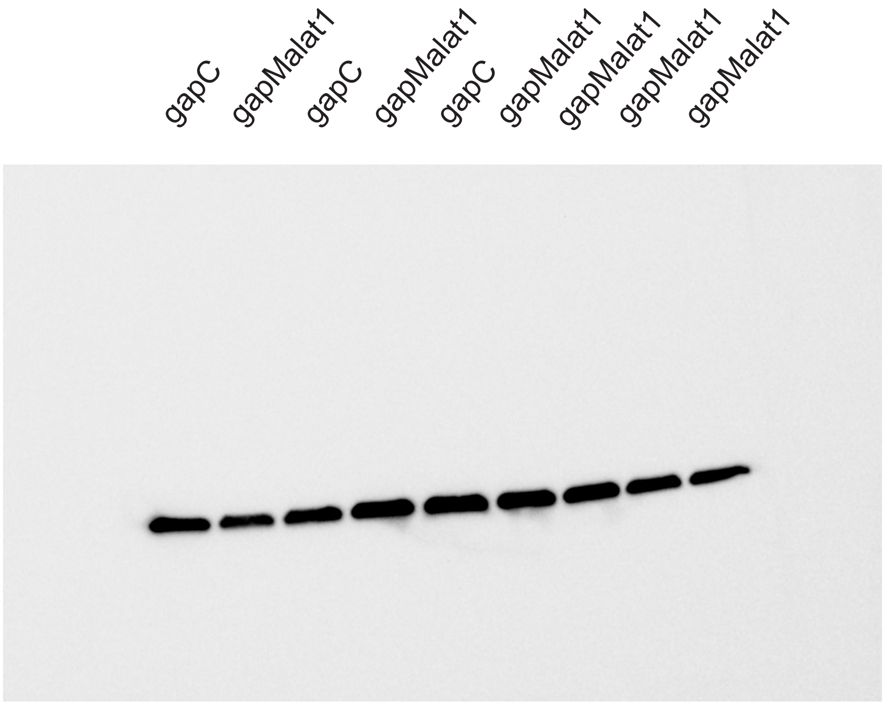

GAPDH

Figure S6. Full blots

Biorad chemidoc Image lab software; original blots for figure 4H

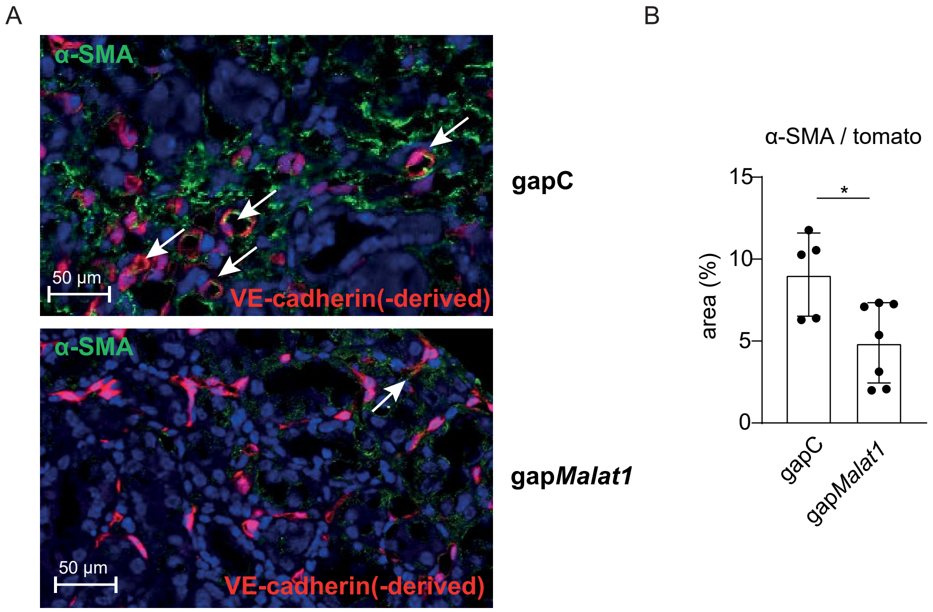

Figure S7. *In vivo Malat1* knockdown reduces endothelial to mesenchymal transition. (A-B) representive images (A) and quantification (B) of co-localization (examples are indicated with arrows) of VE-cadherin-derived tomato positive cells and α-SMA stained positive cells in a UUO kidneys from mice treated with control GapmeR (gapC) or GapmeR against *Malat1* (gap*Malat1*). *P<0.05.

Ingenuity Canonical Pathways	Z-score
Oxidative Phosphorylation	-6.564
Neutrophil Extracellular Trap Signaling Pathway	-4.621
Mitochondrial Dysfunction	4.608
Phagosome Formation	4.536
CREB Signaling in Neurons	4.158
Granzyme A Signaling	4.123
EIF2 Signaling	-3.962
Pathogen Induced Cytokine Storm Signaling Pathway	3.772
S100 Family Signaling Pathway	3.592
FAK Signaling	3.528

Figure S8. Ingenuity Pathway analysis. Top 10 enriched canonical pathways as determined by ingenuity pathway analysis in differentially expressed genes in mouse kidney FACS-sorted ECs from mice treated with *Malat1*-targeting GapmeR**.**

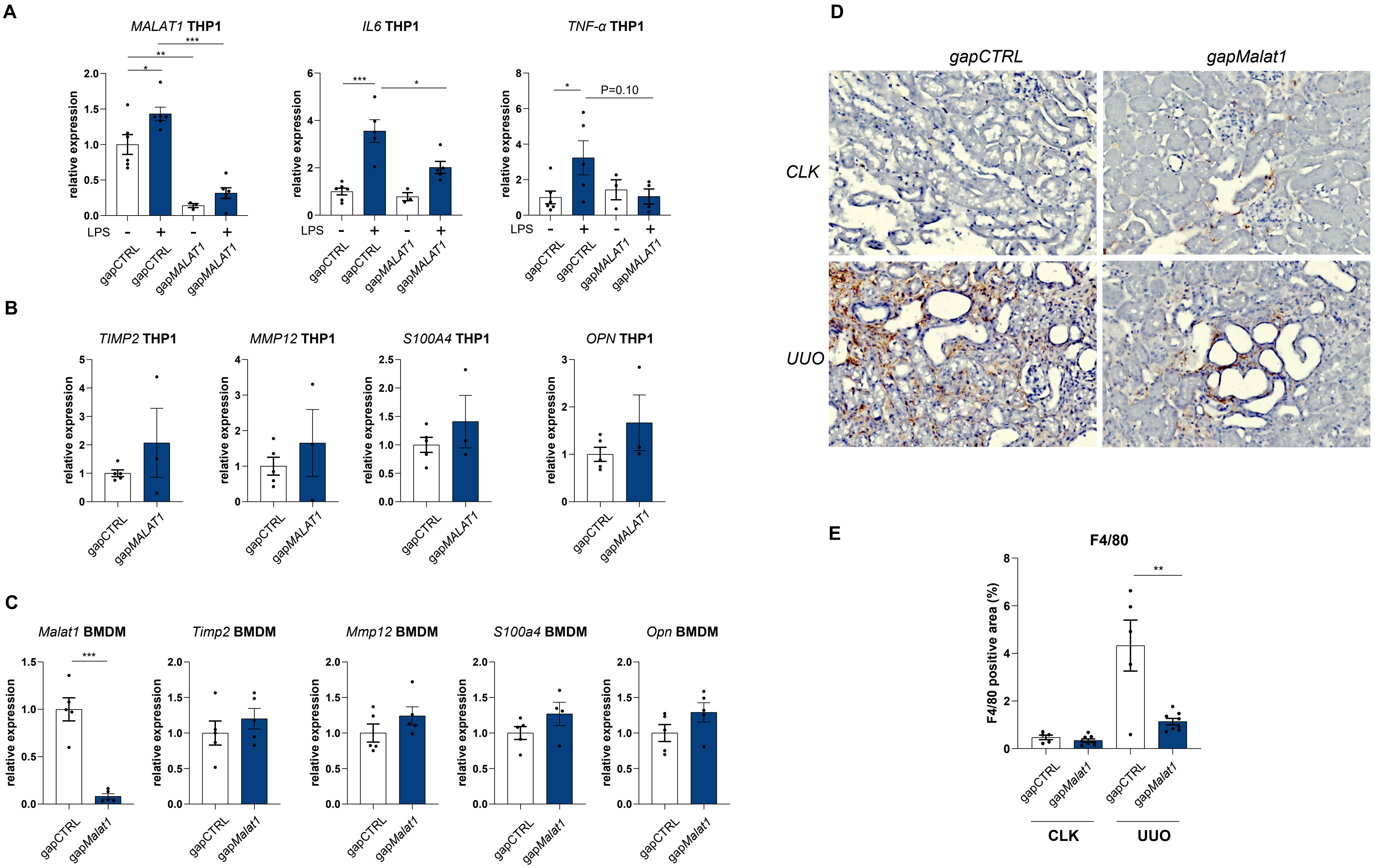

Figure S9. *Malat1* knockdown decreases inflammatory phenotype of macrophages and results in less kidney infiltration of macrophages during injury (A) *MALAT1* expression increases in PMA-differentiated, LPS-stimulated human THP1 cells and is decreased upon gapmer-mediated knockdown, which results in less LPS-induced *IL6* and *TNF-α* expression in THP1 cells. (B) Gene expression of profibrotic macrophage markers. (C) *Malat1* expression decreases upon gapmer-mediated knockdown in mouse bone-marrow derived macrophages (BMDM) and gene expressin of profibrotic macrophage markers in BMDM. (D) Representative microscopic images of kidney F4/80 macrophage staining, and corresponding quantification (E). *P<0.05, **P<0.01, ***P<0.001, CLK = (healthy) contralateral kidney, UUO = unilateral ureteral obstruction.

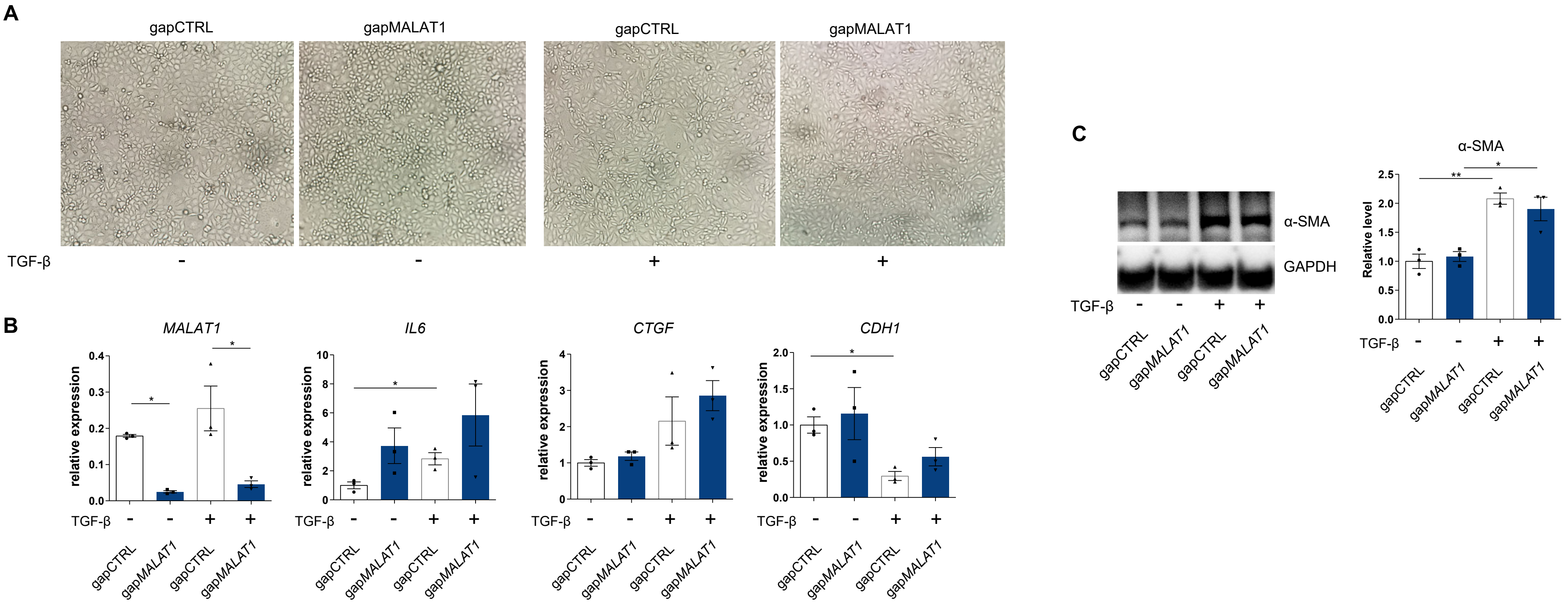

Figure S10. *MALAT1* knockdown in HK2 cells. (A) Representative microscopic images of HK2 epithelial cells with or without TGF-β stimulation and gapmer-mediated knockdown of MALAT1. (B) qRT-PCR plots of *MALAT1, IL6, CTGF and CDH1* expression. (C) Representative western blot for α-SMA and corresponding quantification. *P<0.05, **P<0.01.

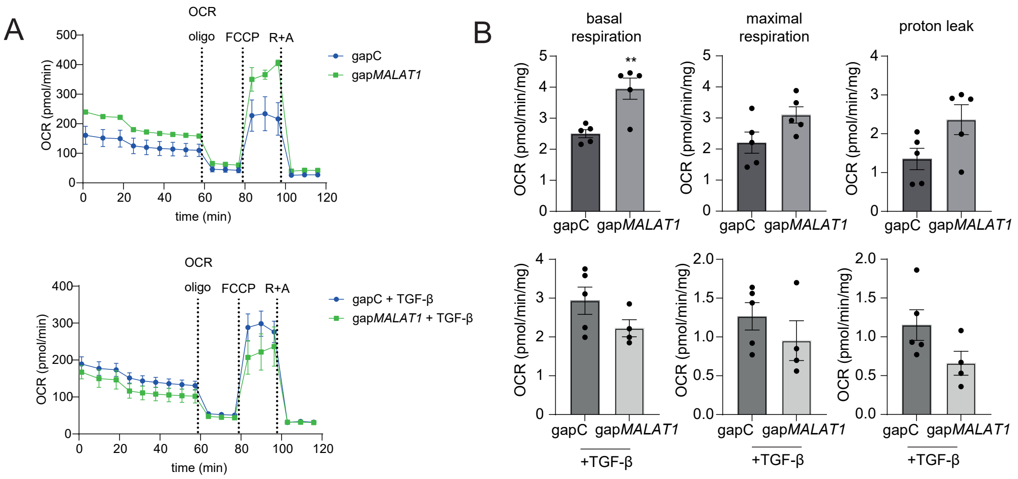

Figure S11. Effect of *MALAT1* knockdown on mitochondrial function. (A-B) Oxygen consumption rate, as determined by the Seahorse assay in HUVECs treated with GapmeR against *MALAT1* (gap*MALAT1*) or GapmeR control (gapC) with (lower panel) or without treatment with TGF-β (upper panel) (A) and corresponding quantification of basal respiration, maximal respiration and proton leak (B). **P<0.01

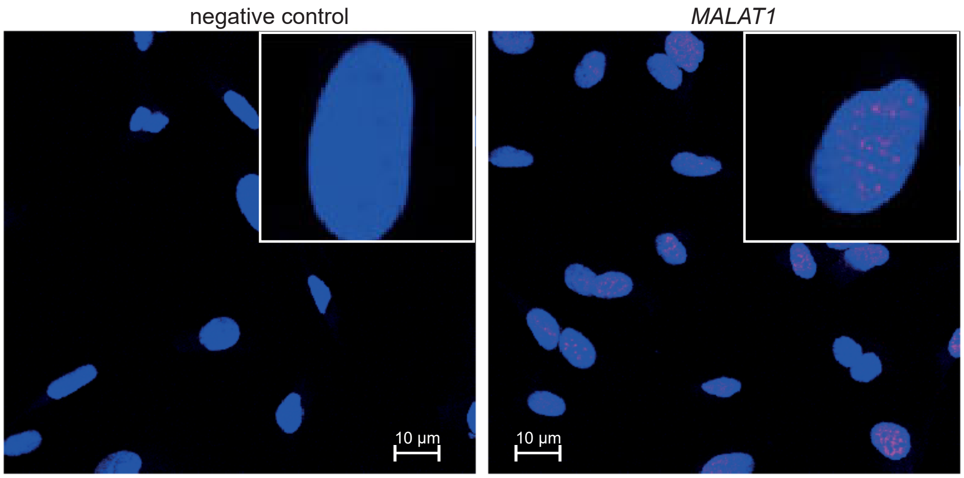

Figure S12. FISH HUVEC. Fluorescent in situ hybridization on HUVECs for *MALAT1* indicates nuclear localization. Upper right panel is zoomed in image of one nucleus.

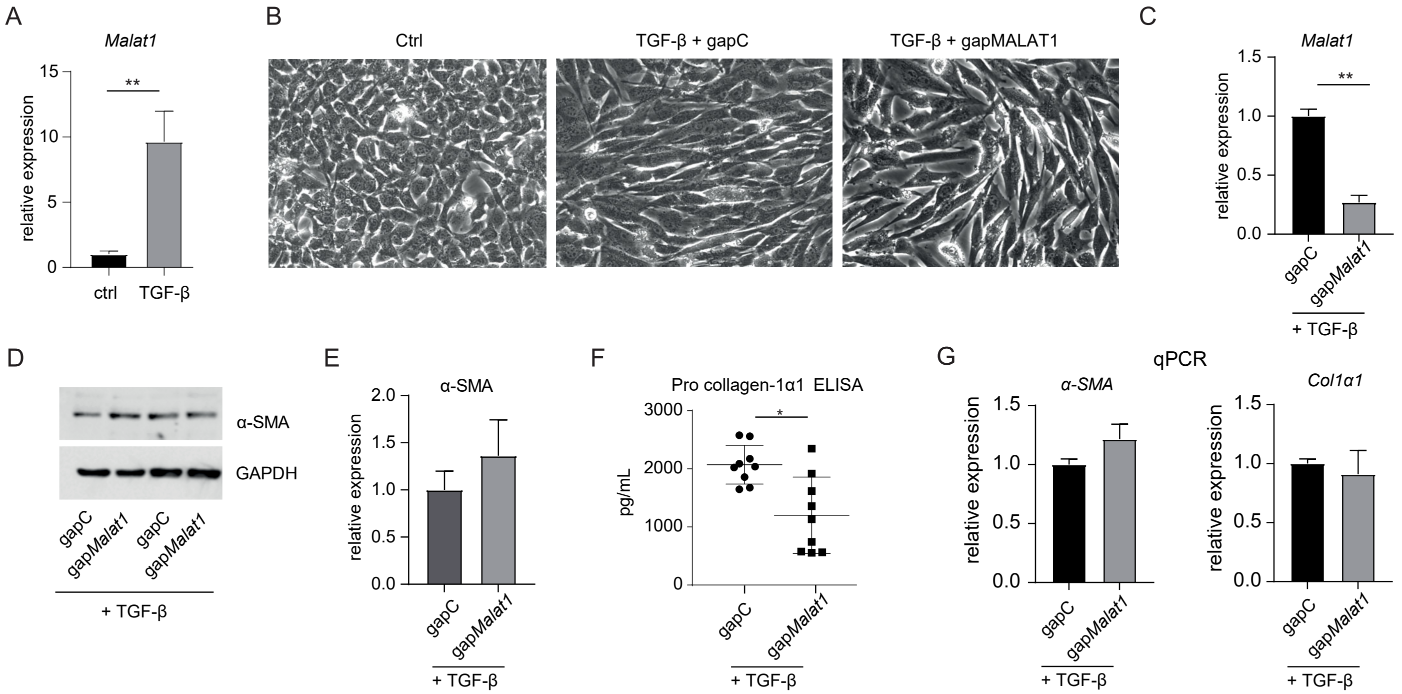

Figure S13. *Malat1* knockdown during endothelial to mesenchymal transition does not alter α-SMA but decreases collagen deposition. (A) *Malat1* expression increases in TGF-β stimulated mouse ECs. (B) Representative microscopic images of mouse ECs that undergo endothelial to mesenchymal transition upon TGF-β treatment, visible from the elongated morphology. (C) *Malat1* was inhibited using GapmeRs and compared to GapmeR control. (D-E) Representative western blots indicate TGF-β induces α-SMA protein expression, while this is not affected by *Malat1* inhibition. (F) *Malat1* knockdown decreased pro collagen1α1 levels as determined by ELISA. (G) Gene expression levels of myofibroblast marker *α-SMA* and fibrotic marker collagen1α1 did not change upon *Malat1* GapmeR. *P<0.05, **P<0.01.

Provided as Supplemental Excel Files:

Table S1. Profiling lncRNAs IRI

Table S2. Profiling lncRNAs UUO

Table S3. Profiling mRNAs IRI

Table S4. Profiling mRNAs UUO

Table S5. RNA-seq EC gapM
