# Supplementary material for: Targeting long non-coding RNA MALAT1 preserves endothelial cell integrity and protects against kidney fibrosis
Source: Mol Ther Nucleic Acids. 2025 Aug 19;36(3):102689. doi: 10.1016/j.omtn.2025.102689 (PMC12445227; doi:10.1016/j.omtn.2025.102689)
Supplement: Document S2. Article plus supplemental information [file mmc3.pdf]

Molecular Therapy
Nucleic Acids
Original Article

# Targeting long non-coding RNA *MALAT1* preserves endothelial cell integrity and protects against kidney fibrosis

Qiao Zhao,[1] Loïs A.K. van der Pluijm,[1] Morgane Gourvest,[3] Atefeh Lafzi,[2] Daniel Peled,[3] Whitney G. Rubin,[3] Juliette A. de Klerk,[1,4] Roderick C. Slieker,[4] Leen M. 't Hart,[4,5] Wendy Stam,[1] Annemarie M. van Oeveren-Rietdijk,[1] Jacques M.G.J. Duijs,[1] Angela Koudijs,[1] Joris I. Rotmans,[1] Hilal Kazan,[6] Anton Jan van Zonneveld,[1] Coen van Solingen,[3] and Roel Bijkerk[1]

[1]Department of Internal Medicine (Nephrology) and the Einthoven Laboratory for Vascular and Regenerative Medicine, Leiden University Medical Center, 2333 ZA Leiden, the Netherlands; [2]Graduate School of Informatics, Department of Health Informatics, Middle East Technical University, Ankara, Turkey; [3]Department of Medicine, Cardiovascular Research Center, New York University Grossman School of Medicine, New York, NY 10016, USA; [4]Department of Cell and Chemical Biology, Leiden University Medical Center, 2333 ZA Leiden, the Netherlands; [5]Department of Biomedical Data Sciences, Section Molecular Epidemiology, Leiden University Medical Center, 2333 ZA Leiden, the Netherlands; [6]Department of Computer Engineering, Antalya Bilim University, Antalya, Turkey

**Loss of integrity of the capillary network is directly associated with the development of kidney fibrosis resulting in chronic kidney disease. Here, we characterized long non-coding RNAs (lncRNAs) in endothelial cells (ECs) during the development of kidney fibrosis. Using a murine EC lineage-tracing model, we observed expression of the conserved lncRNA metastasis-associated lung adenocarcinoma transcript 1 (*Malat1)* to be elevated in ECs upon kidney injury; either by ischemia-reperfusion injury or by unilateral ureteral obstruction (UUO). In addition, we found elevated *MALAT1* expression in the kidney and circulation of patients with fibrotic kidney diseases. Pharmacological intervention of *Malat1* initiated protection against fibrosis in the UUO model, illustrated by a marked decline in collagen deposition and a concomitant decrease in interstitial alpha-smooth muscle actin (α-SMA)-positive cells in the kidney. This protective effect was further highlighted by an increase in capillary density and reduced endothelial-to-mesenchymal transition. Mechanistically, transcriptomic analyses of kidney ECs upon *Malat1* knockdown demonstrated increased EC-matrix-receptor interaction. Furthermore, we show that silencing of *MALAT1* results in increased barrier function and angiogenic response, less vascular leakage, and decreased focal adhesions. Finally, integration with *in silico* analyses and RNA immunoprecipitation confirmed binding of *MALAT1* to SUZ12, a member of the PRC2 complex, suggesting a transcriptional regulatory role for *MALAT1*. Collectively, our findings classify the lncRNA *MALAT1* as an important regulator of EC function and kidney health. As such, targeting *MALAT1* may provide novel strategies to reduce kidney fibrosis.**

## INTRODUCTION

Chronic kidney disease (CKD) has a worldwide prevalence of >10%.[1] Besides its high morbidity, CKD is a leading cause of premature cardiovascular disease.[2] It is estimated that by 2040, CKD will become the 5th leading cause of death, due to the aging population and increased prevalence of non-communicable diseases such as diabetes and hypertension.[3] Irrespective of the etiology, the common pathway in the pathology of CKD involves glomerular sclerosis, tubulointerstitial fibrosis associated with inflammation, myofibroblast proliferation, extracellular matrix accumulation, and tubular atrophy.[4] A central feature of CKD is the progressive loss of the peritubular capillary network, a process that is referred to as rarefaction. Microvascular rarefaction is directly correlated with the severity of fibrosis,[5] and the extent of rarefaction has been found to predict the degree of interstitial damage as well as changes in the glomerular filtration rate in CKD patients.[6] Likewise, emerging evidence indicates that the renal microvascular endothelium of the outer medullary peritubular network is the primary site of injury in kidney allograft nephropathy[7] and that regression of the peritubular network is directly related to a decline in glomerular filtration, interstitial fibrosis, and the severity of chronic allograft nephropathy.[8] These findings suggest an early, rate-limiting role for integrity of the peritubular capillary network in the pathogenesis of kidney fibrosis. Therefore, finding therapeutic strategies to stabilize the microvasculature in patients at risk for progressive renal failure may provide novel treatment options.

Emerging evidence suggests that long non-coding RNAs (lncRNAs) are critical regulators of gene regulatory networks in diverse biological processes, including kidney and endothelial cell (EC) (dys)function.[9,10] lncRNAs are defined as non-coding RNA transcripts longer

Received 19 March 2025; accepted 15 August 2025;
https://doi.org/10.1016/j.omtn.2025.102689.

**Correspondence:** Roel Bijkerk, Ph.D., Department of Internal Medicine (Nephrology) and the Einthoven Laboratory for Vascular and Regenerative Medicine, Leiden University Medical Center, 2333 ZA Leiden, the Netherlands.
**E-mail:** r.bijkerk@lumc.nl


© 2025 The Authors.
Published by Elsevier Inc. on behalf of The American Society of Gene and Cell Therapy.
This is an open access article under the CC BY license (http://creativecommons.org/licenses/by/4.0/).

than 200 nucleotides that exert structural and regulatory functions through their interaction with proteins, DNAs, or RNAs in the nucleus or cytoplasm.[11] lncRNAs have been demonstrated to be involved in gene regulation through a variety of mechanisms, such as epigenetic and transcriptional regulation through their association with chromatin-modifying complexes, regulation of mRNA processing and splicing, and acting as competitive inhibitors of endogenous RNAs (e.g., microRNAs).[11] An example of an lncRNA-directed function in kidney fibrosis can be found in lncRNA *lnc-TSI* that was demonstrated to inhibit renal fibrogenesis by negatively regulating the transforming growth factor β (TGF-β)/Smad3 pathway.[12] Additionally, the lncRNAs RNA imprinted and accumulated in the nucleus and myocardial infarction-associated transcript were shown to mediate myofibroblast formation in kidney fibrosis.[13] lncRNAs are also essential to EC function. For example, lncRNA activated by shear stress in endothelium regulates shear stress sensing and endothelial barrier function through its association with and stabilization of junction proteins, e.g., Platelet endothelial cell adhesion molecule-1 (PECAM-1 and CD31).[14]

In the current study, we aimed to identify lncRNAs that are dysregulated in ECs during kidney fibrosis and to determine whether the modulation of lncRNA expression levels can augment the development of kidney fibrosis. To that end, we used a *Cdh5*-creER;tdTomato mouse model to genetically label ECs and isolated them through fluorescence-activated cell sorting (FACS) from healthy kidneys and injured kidneys that were exposed to unilateral ureteral obstruction or ischemia-reperfusion injury (IRI). Following lncRNA profiling of the sorted cells, we found a signature of differentially expressed lncRNAs in injured cells and demonstrated that lncRNA metastasis-associated lung adenocarcinoma transcript 1 (*MALAT1*) is part of a rate-limiting post-transcriptional network involved in vascular integrity that can potentially be targeted to counteract kidney fibrosis.

## RESULTS

### Kidney fibrosis is associated with loss of vascular integrity and endothelial-to-mesenchymal transition

To allow fate tracing of ECs upon kidney injury, we used a genetic mouse model expressing tamoxifen-inducible Cre driven by the *Cdh5* (VE-Cadherin) promoter (*Cdh5*-Cre-ERT2), as previously described,[15] and crossed these to Rosa-TdTomato$^{fl/fl}$ reporter mice, in which Cre-mediated excision resulted in Tomato expression specific to ECs. Next, we applied the unilateral ureteral obstruction (UUO) model to induce severe kidney fibrosis. In addition, we included the unilateral IRI model as an acute kidney injury model that associates with microvascular injury and develops kidney fibrosis in the longer term. At 10 and 2 days post-intervention, respectively, studies were terminated (Figure 1A). In the UUO model, upon kidney fibrosis, we observed less organized EC-derived Tomato signal (Figure 1B). Notably, several tomato-positive cells appeared elongated and enlarged, suggesting that ECs possibly underwent endothelial-to-mesenchymal transition (EndMT). We costained the Tomato signal with mesenchymal marker alpha-smooth muscle actin (α-SMA) and indeed found several double-positive ECs, confirming that a fraction of ECs underwent EndMT (Figure S1A). We subsequently sorted the Tomato-positive cells using FACS (Figure S1B) and via RT-qPCR confirmed EndMT: acquirement of mesenchymal markers *α-SMA* (*Acta2*) and *Col-1α1* in ECs derived from UUO kidneys compared to ECs derived from healthy kidneys, while EC-markers *VE-cadherin (Cdh5)* and *Pecam-1/Cd31* were downregulated, suggesting loss of EC phenotype (Figures S1C and S1D). Interestingly, in the IRI model, we observed a trend toward increased levels of mesenchymal marker *Col-1α1* in ECs already after 2 days, potentially indicative of an early initiation of the pro-fibrotic phenotype.

### LncRNAs are differentially expressed in ECs during kidney injury

To implicate lncRNAs involved in the injury response of ECs in fibrotic kidney disease, we profiled lncRNAs in FACS-sorted *Cdh5*-derivative ECs isolated from injured and contralateral control kidneys (CLK). These efforts provided an EC-specific lncRNA signature within the *in vivo* kidney injury setting. Differential expression analysis and hierarchical clustering of lncRNA expression revealed clear differential lncRNA expression profile in ECs in diseased kidneys compared to healthy kidneys for both models (Figures 1C and 1D). After IRI, we found 417 lncRNAs to be significantly altered in ECs, of which 280 were decreased and 137 increased. Upon UUO, 587 lncRNAs (322 down and 265 up) were dysregulated in ECs ($-1 < \text{logFC} > 1$, $p < 0.05$; Figure 1E; Tables S1 and S2). Simultaneously, mRNAs were profiled, and subsequent differential expression analysis, hierarchical clustering, and pathway analyses revealed a clear differential mRNA expression profile in ECs in diseased kidneys compared to healthy kidneys (Figures S2A–S2D; Tables S3 and S4). Gene Ontology (GO) biological process analysis of genes differentially expressed in ECs of both IRI and UUO models when compared to CLK indicates "*Actin Cytoskeleton Organization*" and "*Cell-Matrix Adhesion*" to be affected (Figure S2E).

Among differentially expressed lncRNAs, we found 17 lncRNAs that were differentially expressed in both models (Figure 1F). Based on consistent upregulation in both injury models, as well as known conservation between mice and humans and previously reported increased circulating levels in patients with diabetic kidney disease,[16] we hypothesized that lncRNA *Malat1* may play an important role in ECs in kidney fibrosis and was selected to study further. Additionally, transcription factor motif enrichment analysis in the promoter regions of the differentially expressed lncRNAs identified among others HMGA1 to be enriched and to potentially bind the promoter region of *MALAT1* (Figures S3A–S3C). Interestingly, HMGA1 has been linked to regulate EC function via controlling endothelial plasticity and angiogenesis.[17–19] We applied chromatin immunoprecipitation (ChIP) for HMGA1 and confirmed binding to the *Malat1* promoter by this transcription factor (Figure S3D), supporting direct regulation.

### *MALAT1* is increased in human fibrotic kidney disease

To demonstrate relevance for humans, we aimed to determine whether *MALAT1* dysregulation is consistent in human vascular

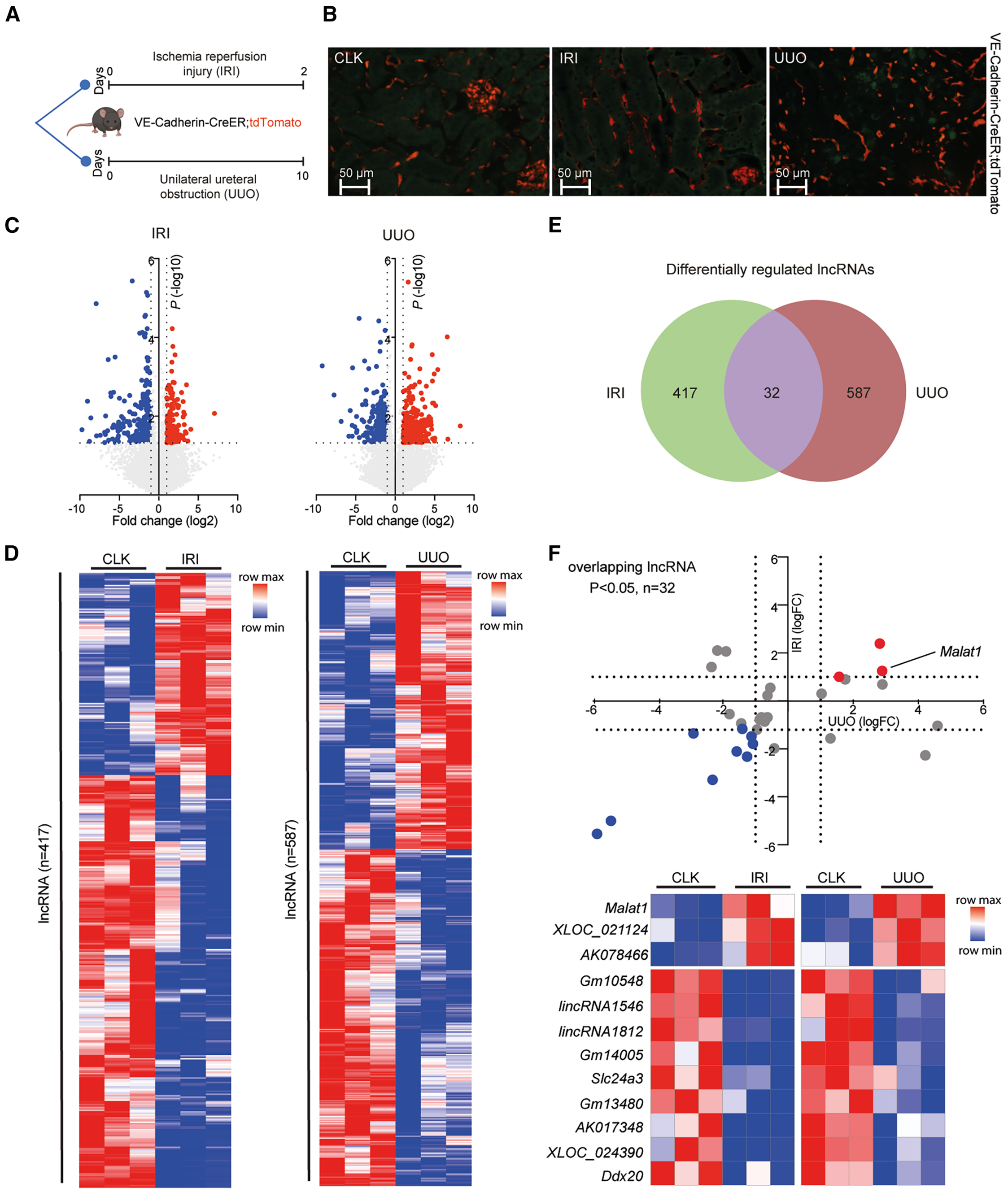


**Figure 1. Loss of vascular integrity during kidney fibrosis and differential lncRNA expression in endothelial cells**

(A) Schematic overview of study setup. (B) Representative images of tdTomato-positive cells (red) in healthy contralateral (CLK), IRI, and UUO kidneys. (C) Volcano plots visualizing differential lncRNA expression between indicated conditions. The blue and red dots correspond to lncRNAs with $p < 0.05$ and $-1$<logFC>1 that are down- or up-regulated, respectively. Gray dots indicate non-significantly changed lncRNAs ($n = 3$ per condition). (D) Hierarchical clustering shows a distinguishable lncRNA expression pattern in VE-cadherin-derived cells in IRI and UUO compared to healthy CLK kidneys ($n = 3$ per condition). (E) Venn diagram indicating number of differentially expressed lncRNAs in IRI and UUO. (F) Scatterplot and heatmap of lncRNAs that are significantly differentially expressed in both models. The blue and red dots correspond to lncRNAs with $p < 0.05$ and $-1$<logFC>1 that are down- or up-regulated, respectively. Gray dots indicate non-significantly changed lncRNAs.

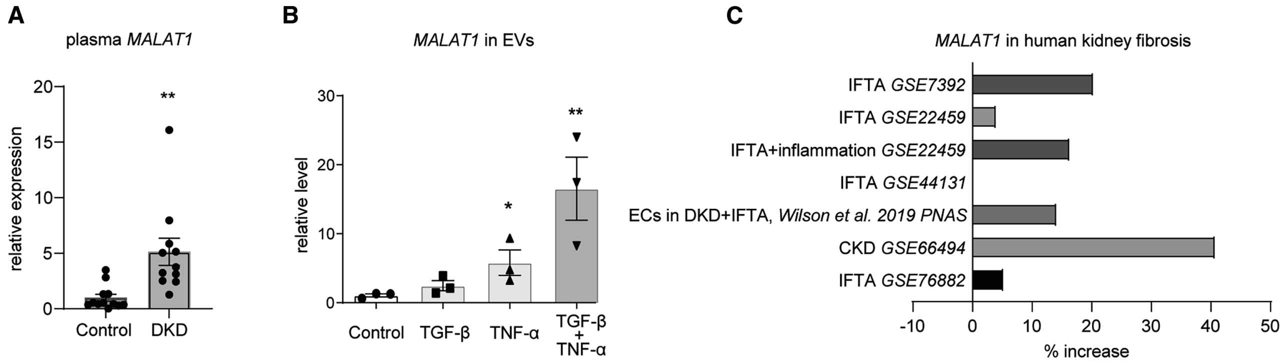


**Figure 2. Increased kidney and circulating *MALAT1* levels in fibrotic kidney disease**

(A) Quantitative reverse-transcription PCR (RT-qPCR) analysis of *MALAT1* levels in plasma of patients with diabetic kidney disease (DKD, $n = 11$) and healthy controls ($n = 12$); student's t test. (B) RT-qPCR analysis of *MALAT1* in extracellular vesicles from HUVECs stimulated with TGF-β and/or TNF-α or vehicle control ($n = 3$); one-way ANOVA. (C) *MALAT1* expression levels in fibrotic kidney tissue compared to controls from different datasets. IFTA, interstitial fibrosis and tubular atrophy; CKD, chronic kidney disease. $^*p < 0.05$ and $^{**}p < 0.01$.

and fibrotic kidney disease. We previously demonstrated that circulating *MALAT1* levels were increased in patients with diabetic kidney disease.[16] We reassessed circulating *MALAT1* levels in the plasma of these patients and confirmed increased *MALAT1* levels when compared to controls (Figure 2A). Since circulating lncRNA levels are often carried in extracellular vesicles (EVs), we hypothesized that activated ECs may secrete increased levels of *MALAT1* in EVs. Indeed, when human umbilical vein endothelial cells (HUVECs) were stimulated with TGF-β and/or tumor necrosis factor alpha (TNF-α), we observed increased *MALAT1* levels in EC-secreted EVs (Figure 2B). Finally, we consulted online available datasets that contained transcriptomics data from human kidneys from patients with fibrotic kidney disease (GSE7392, GSE22459, GSE22459, GSE44131, GSE66494, and GSE76882) and found an upregulation of *MALAT1* in the majority of datasets (Figure 2C), which is consistent with our mouse studies. Importantly, in a single-cell RNA sequencing (RNA-seq) dataset that contained data from ECs in kidneys from patients with diabetes with or without kidney fibrosis,[20] we observed that *MALAT1* is increased in ECs when kidney fibrosis is present (Figure 2C).

### *In vivo* knockdown of *Malat1* reduces kidney fibrosis and preserves vascular integrity

Given the increase of *Malat1* in ECs in UUO and IRI, we hypothesized that knockdown of *Malat1* may protect against disturbed microvascular integrity and the subsequent development of kidney fibrosis. To investigate this, we applied the UUO model in the *Cdh5*-creER; tdTomato mice while inhibiting *Malat1* expression using intraperitoneal injection of *Malat1*-targeting GapmeRs. Silencing of *Malat1* in the kidneys was confirmed by *in situ* hybridization (ISH) for *Malat1* within the kidney tissue (Figures 3A–3C and S4), as well as by RT-qPCR (Figure S5). Interestingly, ISH indicates that, upon UUO, *Malat1* is elevated in the kidney cortex, while gap*Malat1*-mediated knockdown also occurs mainly in the kidney cortex. To assess whether *Malat1* knockdown resulted in decreased kidney fibrosis, we next used a Sirius Red staining to determine collagen deposition (Figure 3D). Indeed, we observed a strong decrease in kidney fibrosis upon inhibition of *Malat1*, amounting to a ∼50% reduction in collagen deposition (Figure 3E). Furthermore, we observed a marked reduction in the number of α-SMA-positive myofibroblasts in kidneys from the gap-*Malat1* mice, compared to kidneys of gapC-treated mice, 10 days after UUO (Figures 3F and 3G). We confirmed this decrease in kidney α-SMA expression upon *Malat1* inhibition using western blot (Figures 3H, 3I, and S6). Given the observed role of *Malat1* in regulating EC function, we then determined the impact of *Malat1* knockdown on renal vascular integrity after UUO. Indeed, we observed that the density of MECA32+ peritubular capillaries in the gap*Malat1*-treated mice was markedly higher than that in the control kidneys (Figures 3J and 3K). Similarly, we observed higher levels of tdTomato+ VE-cadherin(-derived) cells upon *Malat1* knockdown (Figures 3L and 3M). Given that our lineage trace model allows fate tracing of the ECs, we next assessed whether EndMT was also affected by silencing *Malat1*. Indeed, we found decreased numbers of α-SMA-tdTomato double-positive cells, indicating a decrease in EndMT (Figure S7). Taken together, these data further confirm that *Malat1* plays an important function *in vivo* in regulating vascular integrity and thereby in mediating kidney fibrosis.

### Knockdown of *MALAT1* affects cell-cell and cell-matrix interaction in ECs

To understand how depletion of *MALAT1* preserves vascular integrity and decreases fibrosis and myofibroblast formation, we isolated RNA from FACS-sorted VE-cadherin;tdTomato+ cells in the UUO model (10 days) after silencing *Malat1* and performed transcriptomic analysis by RNA-seq. Unsupervised hierarchical clustering of genes differentially expressed in *Malat1*-sufficient and -deficient ECs revealed that *Malat1* significantly reprogrammed transcriptional responses in response to UUO induction (Figure 4A, $-1 < \text{logFC} > 1$, $p < 0.05$; Table S5). In addition, using RT-qPCR, we confirmed knockdown of *Malat1* in the sorted

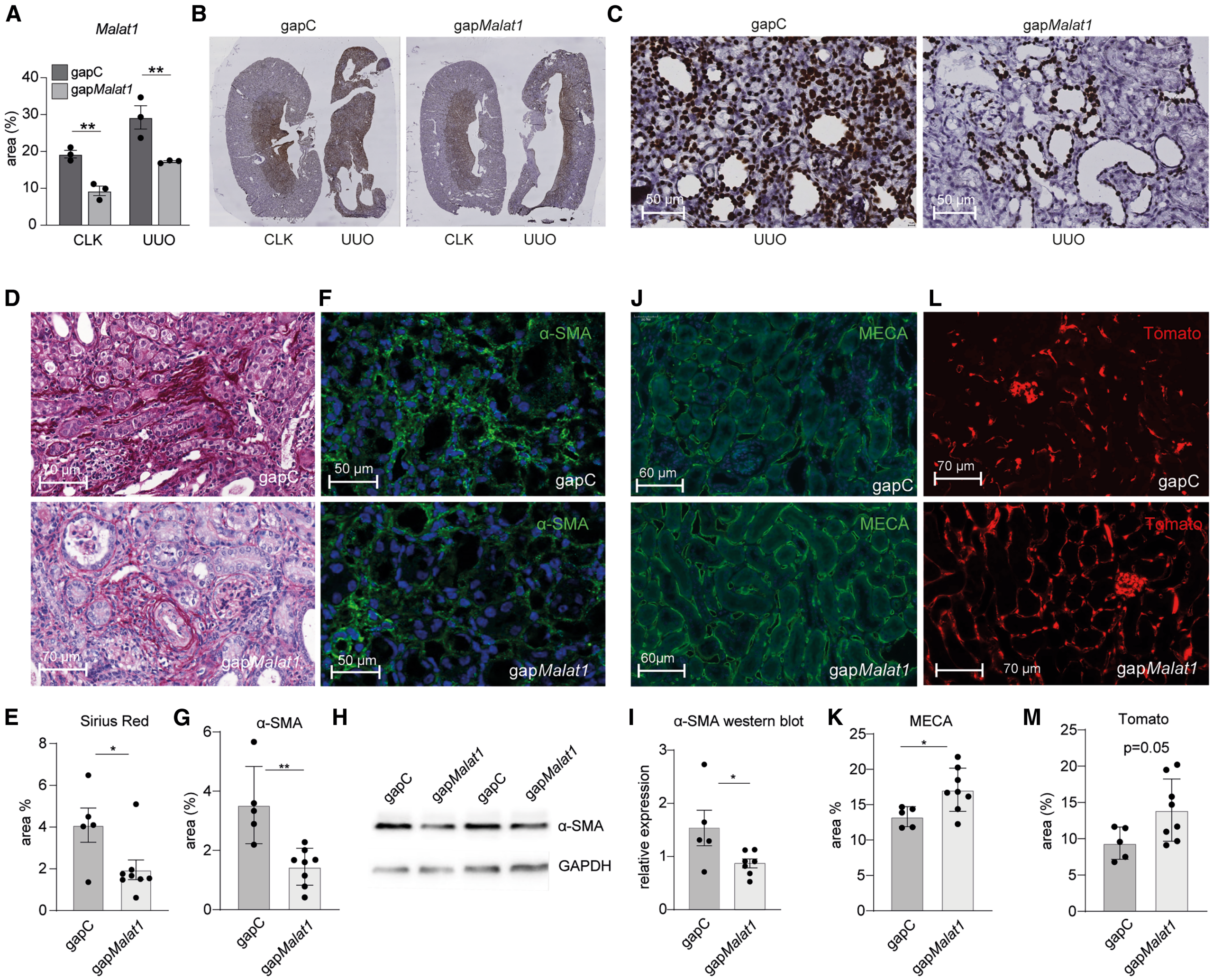


**Figure 3. *In vivo Malat1* knockdown inhibits kidney fibrosis and preserves vascular integrity**
(A) Quantification of *in situ* hybridization for *Malat1* in the kidney upon *Malat1*-targeting GapmeR treatment (gap*Malat1*), compared to control GapmeR (gapC)-treated mice, *n* = 3; one-way ANOVA. (B and C) Representative whole-mount images (B) and zoomed-in images (C) of *in situ* hybridization for *Malat1*. (D and E) Representative images of Sirius Red staining (D) and corresponding quantification (E). (F and G) Representative images of α-SMA staining (F) and corresponding quantification (G). (H and I) Representative western blots for α-SMA (H) and corresponding quantification (I), normalized for glyceraldehyde 3-phosphate dehydrogenase (GAPDH). (J and K) Representative images of endothelial cell marker MECA32 (J) and corresponding quantification (K). (L and M) Representative images of endogenous Tomato label (L) and corresponding quantification (M). For (D)–(M), *n* = 5 (gapC) and *n* = 8 (gap*Malat1*); student's t test. $^*p < 0.05$, $^{**}p < 0.01$, and $^{***}p < 0.001$. CLK, healthy contralateral kidney; UUO, fibrotic kidney from unilateral ureteral obstruction model; gapC, control GapmeR; gap*Malat1*, *Malat1* GapmeR.

VE-cadherin-tdTomato+ cells (Figure 4B). Gene set enrichment analysis of differentially expressed genes identified that "*ECM-Receptor Interaction*" is activated, while metabolic and mitochondrial pathways are suppressed (Figure 4C). In addition, analysis of enriched cellular components indicates that "*Focal Adhesion*" and "*Cell-Substrate Junction*" are affected (Figure 4D). These pathway analyses were confirmed by ingenuity pathway analysis (IPA) that also indicated increased "*Focal Adhesion Kinase (FAK) signaling*," important for cell-matrix interactions, and impaired metabolic pathways, mainly reduced "*Oxidative Phosphorylation*" and increased "*Mitochondrial Dysfunction*" (Figure S8). As such, these data pose two possible mechanisms (cell-matrix/cell-cell interactions and metabolic/mitochondrial function) via which *Malat1* affects EC function and kidney fibrosis.

Before investigating this, we explored the effect of *Malat1* knockdown on macrophages and proximal tubular epithelial cells, as these cells could also be affected by the *Malat1* GapmeRs. By using mouse bone marrow-derived macrophages (BMDMs) and phorbol 12-myristate 13-acetate (PMA)-stimulated human THP-1 cells to induce macrophage phenotype, we found *Malat1* knockdown to induce a modest decrease in inflammatory phenotype, as evidenced

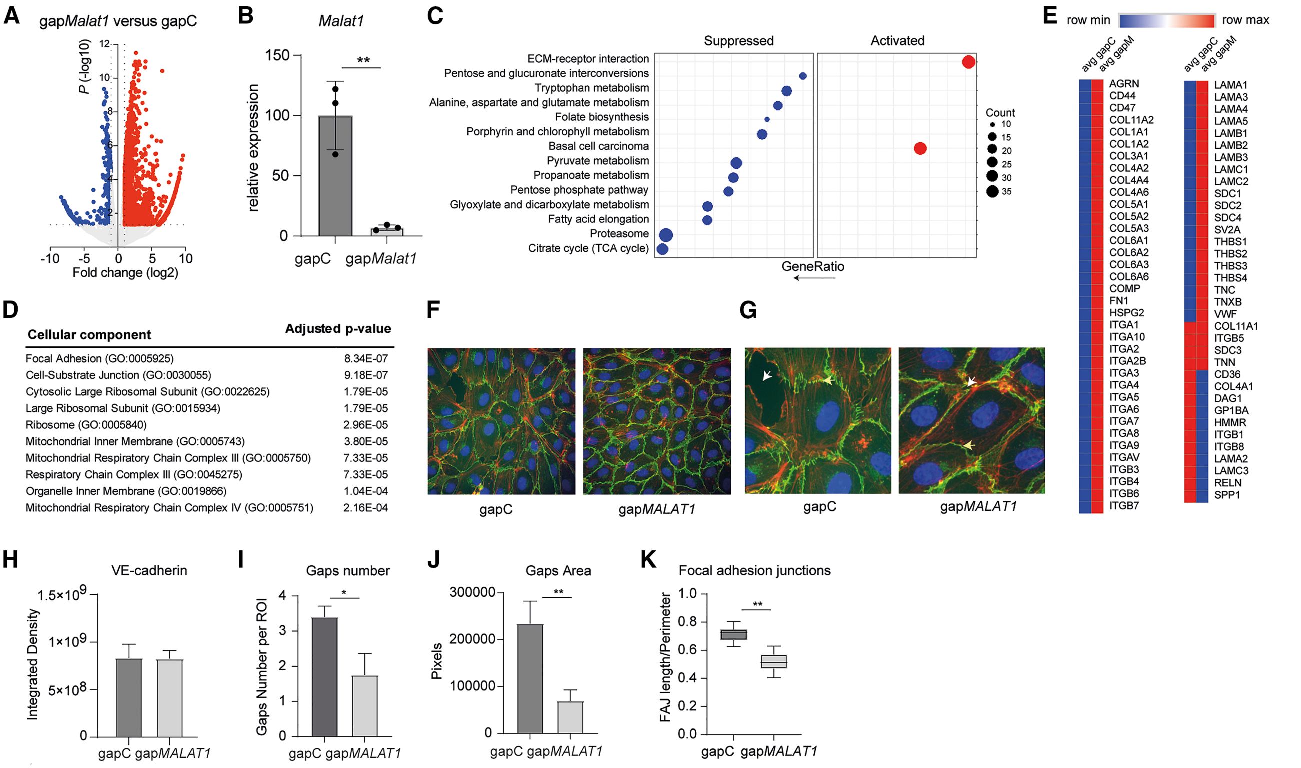


**Figure 4. Knockdown of *MALAT1* affects cell-cell and cell-matrix interaction**

(A) Volcano plots visualizing differential gene expression in ECs *in vivo* upon GapmeR-mediated *Malat1* knockdown. The blue and red dots correspond to mRNAs with $p < 0.05$ and $-1<\text{logFC}>1$ that are down- or up-regulated, respectively. Gray dots indicate non-significantly changed mRNAs, $n = 3$. (B) *Malat1* levels as determined by quantitative reverse-transcription PCR (RT-qPCR) on FACS-sorted mouse kidney ECs; $n = 3$; student's t test. (C and D) Gene set enrichment analyses (C) and cellular component analysis on differentially expressed genes from (A). (E) Heatmap showing relative expression of genes involved in ECM-receptor interaction. (F and G) Representative images of VE-cadherin staining (green) and F-actin (red) (zoomed images in G) on HUVECs treated with gap*MALAT1* or gapC, in the presence of TGF-β. Examples of gaps (loss cell-cell contacts) are shown by white arrows, and focal adhesion junctions by yellow arrows. (H–K) Quantification of total VE-cadherin staining (H), number of gaps (loss of cell-cell contacts) (I), area of gaps (J), and focal adhesion junctions (K); $n = 4$; student's t test. *$p < 0.05$ and **$p < 0.01$.

by gap*MALAT1*-mediated lower *Il6* and *TNF-α* expression upon lipopolysaccharide (LPS) stimulation in PMA-differentiated THP-1 cells (Figure S9A). We next assessed the "pro-fibrotic" phenotype and found no statistically significant alterations, although a trend toward increased expression levels of pro-fibrotic markers was observed in both BMDM and THP-1 cells upon knockdown (Figures S9B and S9C). Then, we assessed kidney macrophage content by F4/80 staining and observed lower number of macrophages in fibrotic kidneys upon *Malat1* knockdown (Figures S9D and S9E). Next, we analyzed the effect of *MALAT1* knockdown on proximal tubular epithelial cells (PTEC) using HK-2 cells. While TGF-β induced a "mesenchymal" pro-fibrotic and pro-inflammatory phenotype, knockdown of *MALAT1* did not affect this phenotype (Figure S10).

To further study the above identified affected pathways in ECs upon *MALAT1* knockdown, we started out with testing the effect of *MALAT1* knockdown in HUVECs on mitochondrial function *in vitro* using Seahorse respirometry. Under basal conditions, *MALAT1* knockdown increased oxygen consumption in ECs (Figure S11A), corresponding to increased basal respiration, maximal respiration, and proton leak (Figure S11B). Upon TGF-β treatment, simulating the *in vivo*-activated state, gap*MALAT1*-treated HUVECs displayed decreased oxygen consumption corresponding to a trend toward decreased basal respiration, maximal respiration, and proton leak (Figures S11A and S11B). While the latter is in line with the RNA-seq data showing decreased mitochondrial function and oxidative phosphorylation, this presumably negative effect may not solely explain the beneficial effects of *Malat1* knockdown *in vivo*. Therefore, we further investigated the role of *MALAT1* in cell-cell and cell-matrix interactions and FAK signaling, as identified under Figures 4C and 4D. When visualizing relative gene expression from genes involved in the ECM-receptor interaction pathway, it is apparent that decreased *Malat1* levels result in higher levels of many of these genes (Figure 4E). Following this notion, we next examined cell-cell and cell-matrix interaction by staining HUVECs for VE-cadherin and F-actin to visualize the actin fibers. While we did not find differences in absolute VE-cadherin staining, a clear decrease in cell-cell contacts was visible upon

measuring the number of gaps between cells and gap area (Figures 4F–4J). More importantly, when analyzing focal adhesion junctions (FAJs), a measure of EC activation and junction remodeling,[21] we found a reduction in these FAJs upon *MALAT1* knockdown (Figure 4K), indeed implicating a more stable cell-matrix interaction, which is in line with Figure 3 demonstrating that *MALAT1* knockdown preserves vascular integrity.

### *MALAT1* potentially acts in *trans* and in *cis*

We next sought to gain more insight into the possible underlying molecular mechanisms of *MALAT1* in EC function. First, we performed fluorescence in situ hybridizationfor *MALAT1* on HUVECs and confirmed nuclear localization (Figure S12). As it was previously indicated that *MALAT1* plays a *cis*-regulatory role in gene transcription,[22] we performed virtual chromosome conformation capture followed by high-throughput sequencing (Hi-C) analysis to identify coregulatory genes and potential in *cis* mechanisms driven by *MALAT1* in its genomic locus.[23] *MALAT1* appears to have a strong association with nearby genes, potentially *FAUP4* and *NEAT1* (Figures 5A and 5B). Increased *Neat1* expression upon *Malat1* knockdown was confirmed in our RNA-seq dataset (Figure 5C). These data suggest a relation between *Malat1* and the genes in its topological associated domain, indicating a potential *cis*-regulatory effect of *Malat1* depletion in kidney ECs. Next, we investigated the potential in *trans* functional roles of *MALAT1.* We identified "*RNA Binding*" to be the top enriched molecular function in our RNA-seq dataset (Figure 5D) and found a strong overlap with genes involved in RNA binding by chromatin-associated proteins (Figure 5E).[24,25] These data prompted us to analyze the presence of potential RNA-binding domains in *MALAT1*, and we found a strong enrichment of the PF00076 RNA recognition motif (Figure 5F). Using CatRAPID, we subsequently found numerous RNA-binding proteins that are predicted to directly interact with *MALAT1* and *Malat1* (Figure 5G). Most strikingly, we found polycomb protein SUZ12 to be the top hit, while ChIP-X Enrichment Analysis on the differentially expressed genes from our RNA-seq indeed identified SUZ12 and enhancer of zeste homolog 2 that together with SUZ12 forms the polycomb repressive complex 2 (PRC2), to be the strongest associated transcription factors (Figure 5H). These findings prompted us to investigate direct binding of *MALAT1* to SUZ12. To that end, we assessed whether *MALAT1* associated with SUZ12 by performing RNA immunoprecipitation. Indeed, qPCR of SUZ12 immunoprecipitates showed enrichment of *MALAT1* when compared to control immunoglobulin G (IgG) immunoprecipitates (Figure 5I). This interaction of *MALAT1* has been previously confirmed in other cell types[26,27] and suggests that the nuclear *MALAT1* may be involved in ECs in in *trans* chromatin binding and transcriptional regulation via SUZ12 and the PRC2.

### *In vitro* knockdown of *MALAT1* inhibits EC activation

Given our findings that *MALAT1* inhibition preserves vascular integrity and affects cell-cell/cell-matrix contacts, we next set out to determine whether *MALAT1* knockdown indeed impacts EC and vascular function. First, as we observed EndMT to occur during kidney fibrosis, we used our *in vitro* mouse EC model for EndMT[28] to investigate the role of *MALAT1* and showed that TGF-β induces *Malat1* expression (Figure S13A). However, *Malat1* knockdown did not prevent the TGF-β induced elongation of the cells associated with EndMT (Figures S13B and S13C), nor the increase in mesenchymal marker α-SMA (Figures S13D and S13E). *Malat1* silencing decreased secreted pro-collagen1α1 levels (Figure S13F), while *α-SMA* and *col1α1* gene expression were not affected (Figure S13G). These data suggest that reducing cellular *Malat1* levels may not affect the cellular transition but could influence pro-fibrotic properties. Next, we knocked down *MALAT1* in HUVECs using GapmeR antisense oligonucleotides (gap*MALAT1*) or control GapmeRs (gapC) and performed a series of functionality assays. First, we assessed the angiogenic response of HUVECs treated with gap*MALAT1* in the Organoplate microfluidic system-based angiogenesis assay and found that silencing of *MALAT1* resulted in less angiogenesis (Figure 6A) as shown by reduced sprout length and area (Figures 6B and 6C). Then, the endothelial barrier function was assessed by trans-endothelial electrical resistance measurements of HUVECs. Silencing of *MALAT1* expression increased the capacity of HUVECs to form a tight barrier (Figure 6D). In line with the data provided in Figure 4, application of mathematical modeling (provided by the ECIS software) indicated that the increased barrier function upon knockdown of *MALAT1* was driven by significantly enhanced cell-cell contacts as well as a trend toward more efficient cell-matrix contacts (Figure 6E). Since a tight barrier prevents vascular leakage, we leveraged the Organoplate microfluidic system[29] to test the effect of *MALAT1* depletion on the ability of HUVECs to form a leak tight 3D capillary-like vessel. Perfusion of these capillary-like vessels with fluorescently labeled albumin allows measurement of its potential leakage through the vessel (Figure 6F). We observed that *MALAT1* knockdown in HUVECs leads to decreased vascular leakage when compared to gapCTRL (Figures 6G and 6H). Collectively, *MALAT1* emerges to be an important regulator in driving EC integrity/barrier function and activation.

## DISCUSSION

In this study, we demonstrate that lncRNA *MALAT1* is strongly increased in ECs during the fibrotic response upon kidney injury. Knockdown of *Malat1* inhibited EC activation and resulted in preserved vascular integrity in the kidney and decreased kidney fibrosis Together, our findings support a facilitatory role for *MALAT1* negatively impacting vascular integrity in reverberating kidney fibrosis.

Cell-matrix and cell-cell interactions are vital to ECs to maintain stable barrier function and integrity of the blood vessels.[30] We found that knockdown of *MALAT1*, using antisense oligonucleotides *in vitro*, increased barrier function, decreased vascular leakage, preserved cell-cell contacts, and decreased the formation of FAJs, all features of activation and remodeling of ECs.[21] In addition, we observed decreased angiogenesis, which corroborates with previous reports showing that depletion of *MALAT1* inhibits the angiogenic response of ECs,[31,32] as well as other studies demonstrating a pathogenic role of *MALAT1* in EC dysfunction.[33,34] Given the increase in *MALAT1* levels upon kidney injury both in humans and mice, these data clearly point toward a role for *MALAT1* in activation of ECs and

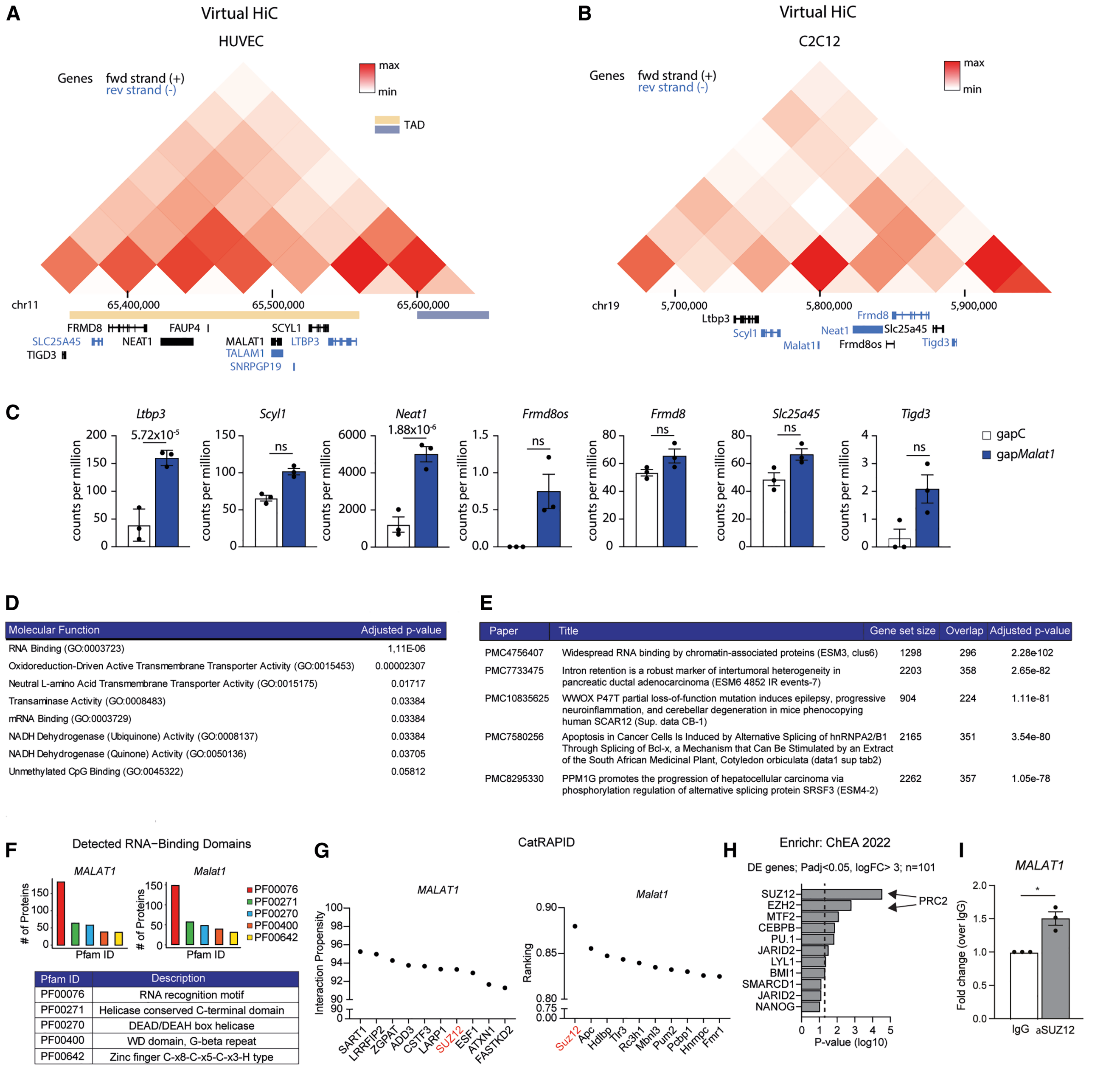


| Molecular Function | Adjusted p-value |
|---|---|
| RNA Binding (GO:0003723) | 1,11E-06 |
| Oxidoreduction-Driven Active Transmembrane Transporter Activity (GO:0015453) | 0.00002307 |
| Neutral L-amino Acid Transmembrane Transporter Activity (GO:0015175) | 0.01717 |
| Transaminase Activity (GO:0008483) | 0.03384 |
| mRNA Binding (GO:0003729) | 0.03384 |
| NADH Dehydrogenase (Ubiquinone) Activity (GO:0008137) | 0.03384 |
| NADH Dehydrogenase (Quinone) Activity (GO:0050136) | 0.03705 |
| Unmethylated CpG Binding (GO:0045322) | 0.05812 |

| Paper | Title | Gene set size | Overlap | Adjusted p-value |
|---|---|---|---|---|
| PMC4756407 | Widespread RNA binding by chromatin-associated proteins (ESM3, clus6) | 1298 | 296 | 2.28e-102 |
| PMC7733475 | Intron retention is a robust marker of intertumoral heterogeneity in pancreatic ductal adenocarcinoma (ESM6 4852 IR events-7) | 2203 | 358 | 2.65e-82 |
| PMC10835625 | WWOX P47T partial loss-of-function mutation induces epilepsy, progressive neuroinflammation, and cerebellar degeneration in mice phenocopying human SCAR12 (Sup. data CB-1) | 904 | 224 | 1.11e-81 |
| PMC7580256 | Apoptosis in Cancer Cells Is Induced by Alternative Splicing of hnRNPA2/B1 Through Splicing of Bcl-x, a Mechanism that Can Be Stimulated by an Extract of the South African Medicinal Plant, Cotyledon orbiculata (data1 sup tab2) | 2165 | 351 | 3.54e-80 |
| PMC8295330 | PPM1G promotes the progression of hepatocellular carcinoma via phosphorylation regulation of alternative splicing protein SRSF3 (ESM4-2) | 2262 | 357 | 1.05e-78 |

| Pfam ID | Description |
|---|---|
| PF00076 | RNA recognition motif |
| PF00271 | Helicase conserved C-terminal domain |
| PF00270 | DEAD/DEAH box helicase |
| PF00400 | WD domain, G-beta repeat |
| PF00642 | Zinc finger C-x8-C-x5-C-x3-H type |

**Figure 5. Virtual Hi-C analysis indicates possible coregulation with *NEAT1*, while *MALAT1* directly binds SUZ12**

(A and B) Virtual Hi-C analysis in HUVECs (A) and mouse C2C12 (B) within the 300 kb region of *MALAT1* or *Malat1*, respectively. (C) Expression of Malat1 neighboring genes in sorted mouse kidney endothelial cells after gapMalat1 treatment or gapC, $n = 3$. (D) Gene set enrichment analysis for enriched molecular functions based on differentially expressed genes in sorted endothelial cells from mouse kidneys upon *Malat1* knockdown, compared to control. (E) "Rummagene" analysis compared differentially expressed genes with other datasets. In parentheses the specific subset of data is indicated that overlaps. (F) RNA-binding domain prediction analysis of both human and mouse *MALAT1*. (G) CatRAPID analysis of both human and mouse *MALAT1*. (H) ChEA analysis on differentially expressed genes in sorted mouse kidney endothelial cells after gapMalat1 treatment or gapC. (I) SUZ12 RNA immunoprecipitation in HUVECs demonstrates direct binding of *MALAT1* to SUZ12; $n = 3$; student's t test. $^{*}p < 0.05$.

loss of vascular integrity that may be averted by using GapmeRs *in vivo* to inhibit *MALAT1*. Indeed, we show in the UUO model that this approach results in silencing of *MALAT1* followed by preserved kidney vascular integrity and suppression of kidney fibrosis.

To gain more insight into *MALAT1*'s in *cis* and in *trans* molecular mechanisms, we performed *in silico* analyses on our *in vivo* transcriptomics data and identified a potential regulatory role for *MALAT1* via SUZ12, a member of the repressive complex PRC2,

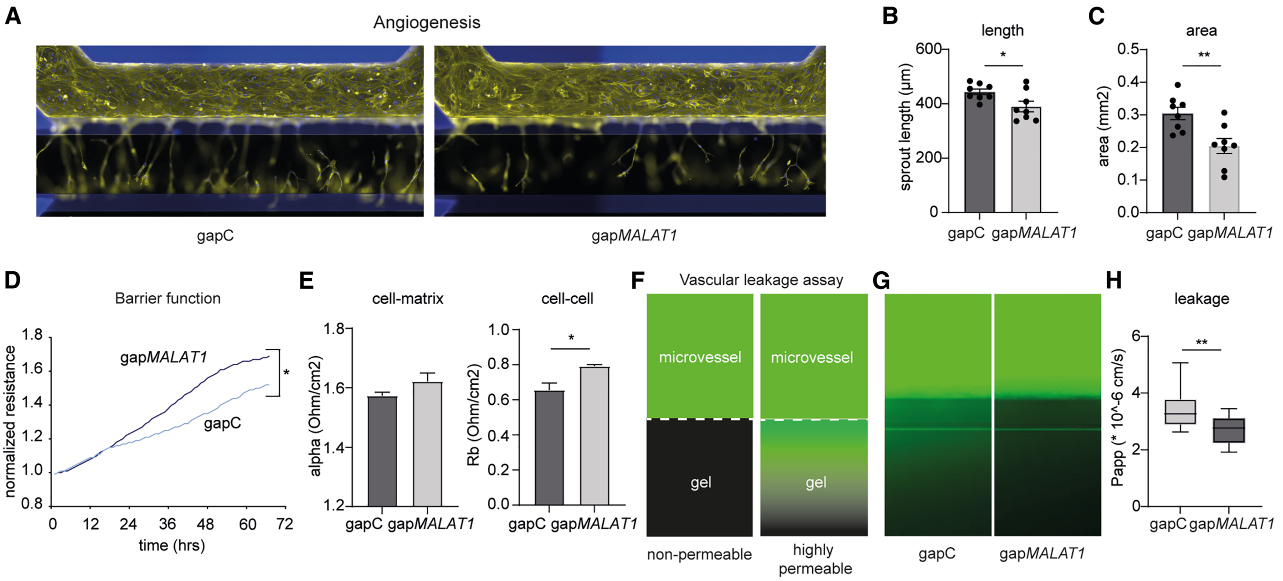


**Figure 6. Knockdown of *MALAT1* in ECs increases barrier function and reduces angiogenesis**

(A–C) Representative images (A) of vascular endothelial growth factor (VEGF)-, basic fibroblast growth factor (bFGF)-, and sphingosine-1-phosphate (S1P)-induced angiogenesis upon knockdown of *MALAT1* (gap*MALAT1*) or control (gapC) and corresponding quantification of average sprout length (B) and total area (C); $n = 8$; student's t test. (D and E) Trans-endothelial electrical resistance of ECs after treatment with gap*MALAT1* or gapC over time (D), attributable to cell-matrix contacts (alpha) and cell-cell contacts (Rb) (E); $n = 4$; student's t test. (F) Schematic representation of the leakage assay, with HUVEC-based 3D capillary-like vessels in the upper perfusion channel, separated from a collagen gel in the lower channel with a phaseguide. Leak tight vessels have limited leakage of fluorescently labeled albumin, while increased permeability of the vessels results in increased fluorescent signal in the gel channel. (G and H) Analysis of leakage assay after knockdown of *MALAT1* (gap*MALAT1*) or control (gapC) in HUVECs presented in (F), $n = 5$. Representative photographs (G) and quantification of the permeability of capillary-like vessels (H). $^*p < 0.05$ and $^{**}p < 0.01$.

which is important in chromatin regulation and transcriptional repression.[35] Supporting this notion, it has indeed been previously demonstrated that *MALAT1* can directly bind SUZ12[26] and is important in transcriptional regulation via chromatin interactions.[27,36,37] Interestingly, it was also described that co-analysis of data from capture hybridization analysis of RNA targets (CHART) and ChIP followed by sequencing showed that *MALAT1* occupies the same sites as PRC2 throughout the mouse and human genomes.[38] Importantly, we validated this direct physical interaction of SUZ12 and *MALAT1* in HUVECs using RNA immunoprecipitation. Moreover, PRC2 has been found to decrease integrin expression in ECs and is important in maintenance of vascular health,[39,40] while a strong link has been found between VE-cadherin expression and PRC2/SUZ12-mediated regulation of an EC-transcriptional program involved in vascular stability.[41] As such, a picture emerges that *MALAT1* is involved in chromatin regulation and transcription via binding SUZ12 leading to repression of a transcriptional program involved in maintaining EC function, possibly in conjunction with its neighboring lncRNA *NEAT1*,[27,42] that our data suggest are co-regulated.

While many studies also describe a role for *MALAT1* as competing endogenous RNA for microRNA, including in the setting of kidney fibrosis,[42,43] our data suggest that *MALAT1* functions as a transcriptional regulator. The subcellular localization of *MALAT1* that we, and others,[27,44] confirmed to be prominent in the nucleus, suggests that *MALAT1* functions in the nucleus and has a less significant role in sponging (cytoplasmic) microRNAs. In addition, since *MALAT1* localizes to the nucleus, we used a GapmeR-based strategy of targeting *MALAT1*, as GapmeRs are designed and shown to reach the nucleus, while short hairpin RNAs mediate RNA degradation primarily in the cytoplasm. In light of these differences in targeting, this may result in different mechanisms of action yet providing protection against kidney fibrosis.[43]

While we find a clear effect of *MALAT1* knockdown on EC function, it cannot be excluded that the protective effect that is observed by *MALAT1* silencing *in vivo* is also mediated through other cell types. For example, *MALAT1* regulates differential activation of macrophages[45] and mediates podocyte injury[46] that may contribute to the development of kidney fibrosis. Indeed, we found that *MALAT1* knockdown results in a less inflammatory and potentially pro-fibrotic phenotype, in line with previous findings.[45] This combined anti-inflammatory, pro-fibrotic macrophage phenotype as a result of *Malat1* knockdown was previously shown to induce fibrosis in the setting of lung fibrosis[45] and would therefore not explain our protective findings in kidney fibrosis, making it likely that the observed lower number of renal macrophages is the consequence of less injury. Yet, a protective effect based on the less inflammatory phenotype cannot be excluded. In addition, *MALAT1* is also highly expressed in tubular epithelial cells, where we, upon GapmeR treatment, also observe knockdown using ISH. However, we found no

effect of *MALAT1* knockdown on the pro-fibrotic phenotype of HK-2 cells, in line with previous findings that *MALAT1* silencing in HK-2 kidney epithelial cells did not result in any functional alterations but mainly altered EC function,[47] suggesting that tubular epithelial cells are not implicated in mediating the protective effects of *Malat1* knockdown but hinting at at least a partial regulatory function for *Malat1* in ECs. Surprisingly, genetic *Malat1* deletion in this study did not result in any antifibrotic effect in the renal IRI model.[47] This may be explained by the fact that, besides being a different injury model, full knockout of a lncRNA may yield different results than pharmacological knockdown; i.e., it has been demonstrated that knockout of *Malat1* has no major phenotypes in mice,[22,48] however, it does affect several physiological and pathophysiological processes in adult mice. Moreover, compensatory mechanisms may be in play as lncRNAs often function as rheostats, suggesting pharmacological targeting of *Malat1* may be more relevant in the setting of kidney fibrosis. Yet, it should be noted that it cannot be excluded that GapmeR-mediated knockdown can result in off-target effects.

Next to increased levels of *MALAT1* in mouse kidneys, we observed upregulation of (endothelial) *MALAT1* in human fibrotic kidney disease. Furthermore, we validated that circulating levels of *MALAT1* are increased in human fibrotic kidney disease, which is in line with previous reports.[16,47,49] Interestingly, since most circulating lncRNAs are carried by EVs, it is tempting to speculate that ECs may be responsible for increased secretion of EVs containing *MALAT1*, especially as it has been demonstrated that, upon kidney injury, more activated EC-derived EVs appear in the circulation.[50,51] Moreover, high glucose levels increase total HUVEC-derived EVs.[52] Following this, our *in vitro* studies indicate that activated ECs secrete more EV-carried *MALAT1*, suggesting that this may indeed be the reason for the higher circulating *MALAT1* levels as observed in patients with kidney disease.

Taken together, our studies identify *MALAT1* as an important regulator of EC function potentially driving kidney fibrosis. Silencing *MALAT1* reduced fibrosis by preserving vascular integrity, and therapies aimed at inhibiting *MALAT1* in the vasculature may serve as potential treatment for CKD.

## MATERIALS AND METHODS

### Animals

All animal experiments were approved by the animal welfare committee of the veterinary authorities of the Leiden University Medical Center. Standard chow diet and drinking water were provided ad libitum. Eight-week-old male B6.Cdh5-Cre-ERT2;tdTomato mice (*Cdh5*-creER;tdTomato) were used, in which tamoxifen-inducible Cre-mediated excision results in endothelial Tomato expression. Mice received intraperitoneal 2 mg/0.2 mL tamoxifen for 5 consecutive days. The UUO model was performed through a left flank incision, followed by identification of the ureter and double ligation thereof close to the lower pole of the kidney with two separate silk ties. After 10 days, the mice were killed. For the *Malat1* GapmeR experiment, locked nucleic acid (LNA) control GapmeR or LNA *Malat1* GapmeR (20 mg/kg; Exiqon) were injected intraperitoneally twice: 2 days before and 2 days after UUO surgery. The unilateral IRI model was performed via an abdominal incision, after which the renal artery and vein were identified and, using surgical clamps (S&T, Neuhausen, Switzerland), unilaterally clamped for 45 min. The contralateral kidneys were used as controls. After 2 days, the mice were killed. Kidneys were removed, and RNA and protein were isolated as described in the following.

### lncRNA and mRNA profiling

Profiling of lncRNAs and mRNAs was performed by Arraystar Inc. according to protocol using the Agilent Array platform. Sample preparation and microarray hybridization were based on standard protocols of the manufacturer with minor modifications. In brief, mRNA was purified from total RNA after rRNA removal (mRNAONLY Eukaryotic mRNA Isolation Kit, Epicentre). Each sample was then amplified and transcribed into fluorescent cRNA along the full length of transcripts without bias using a mix of oligo(dT) and random primers (Arraystar Flash RNA Labeling Kit, Arraystar). Labeled cRNAs were then hybridized on the "Mouse lncRNA Array v.3.0" (8 K $\times$ 60 K, containing 35.923 lncRNAs and 24.881 coding transcripts, Arraystar). After washing the slides, the arrays were scanned on Agilent Scanner G2505C. Agilent Feature Extraction software (version 11.0.1.1) was used for analysis of acquired array images. GeneSpring GX v.12.1 software package (Agilent Technologies) was used to perform quantile normalization and data processing. Following quantile normalization of raw data, lncRNAs and mRNA with at least 6 out of 12 samples having flags in present or marginal ("All Targets Value") were selected for further data analysis. Statistically significant differentially expressed lncRNAs and mRNAs between the two groups were identified through volcano plot filtering.

### Cell culture

Primary HUVECs were isolated from human umbilical cords as previously described[53] and cultured on 1% gelatin-coated surfaces in endothelial cell growth medium 2 (C-39216, PromoCell, Germany) supplemented with antibiotics. Where indicated, HUVECs were treated with 10 ng/mL TGF-β1 and/or 10 ng/mL TNF-α (Sigma). Mouse embryonic endothelial cells (MEECs), as previously described,[28] were cultured on 1% (w/v) gelatin coating in Dulbecco's modified Eagle's medium (Gibco/Invitrogen, Breda, the Netherlands) supplemented with 10% fetal calf serum and 2 mM L-glutamine (Invitrogen). MEECs were treated for 48 h with 2 ng/mL TGF-β3 (Peprotech, London, UK). The human PTEC cell line (HK-2 cells) was grown in serum-free DMEM/HAM-F12 medium (Bio-Whittaker, Walkersville, MD) supplemented with 100 U/mL penicillin, 100 μg/mL streptomycin (Invitrogen, Breda, The Netherlands), 1$\times$ insulin-transferrin-selenium, 40 ng/mL triiodothyronine, 10 ng/mL epidermal growth factor, 36 ng/mL hydrocortisone, triiodothyronine (40 ng/mL), epidermal growth factor (10 ng/mL), and hydrocortisone (36 ng/mL) (all from Sigma, Zwijndrecht, The Netherlands). HK-2 cells were stimulated with 10 ng/mL TGF-β1 (PeproTech). BMDMs were prepared through flushing bone marrow from tibiae and femora of 6- to 8-week-old mice. Cells were

differentiated into macrophages in DMEM medium supplemented with 10% FBS, 1% P/S, and 15% L929-conditioned media for 7 days. THP-1 cells were obtained from ATCC (TIB202) and cultured in 10% FBS-supplemented RPMI1640 medium with 0.01 μg/mL L-glutamine, penicillin, and streptomycin, and 0.05 mM 2-mercaptoethanol was added. To differentiate THP-1 cells from THP-1 macrophages, 100 nM PMA (Sigma-Aldrich) was used. After 3 days, the PMA-containing medium was replaced by normal growth medium, and the cells were used for experiments where 100 ng/mL of LPS was used. Cells were transfected at 60%–75% confluence with 50–100 nM LNA GapmeR (Exiqon, Vedbaek, Denmark) targeting *MALAT1/Malat1* or control GapmeR using Lipofectamine 3000 (Life Technologies, Carlsbad, CA) according to the manufacturer's protocol.

#### FACS

Kidneys were extracted from three individual mice per injury model and mechanically dissociated and filtered through 100 and 40 μm filters. The obtained cell suspension was then sorted on the FACSAria II (BD Biosciences, Franklin Lakes, NJ, United States).

Cell debris was gated out using an FSC-A/SSC-A plot (gate 1), doublets were gated out using an SSC-W/SSC-H plot (gate 2) and an FSC-W/FSC-H plot (gate 3), and these three gates were combined. Next, from the combined exclusion gate, cells that were positively identified having a fluorescent tomato signal were collected.

#### RNA isolation and RT-qPCR analysis

Total RNA was isolated using Trizol reagent (Invitrogen) combined with the RNeasy Micro Kit (QIAGEN, The Netherlands) and reverse transcribed using iScript (Bio-Rad) according to the manufacturers protocol. RT-qPCR of target genes was performed using SYBR Green Master Mix (Applied Biosystems). Used primer sequences of target genes were mouse α-SMA (sense) CGTGGCTATTCCTTCGTGAC; mouse α-SMA (antisense): GCGTTCGTAGCTCTTCTCC; mouse Col1α1 (sense): TGACTGGAAGAGCGGAGAGT; mouse Col1α1 (antisense): GTTCGGGCTGATGTACCAGT; mouse β-actin (sense): AGGTCATCACTATTGGCAACGA; mouse β-actin (antisense): CCAAGAAGGAAGGCTGGAAAA; human IL6 (sense):

ACTCACCTCTTCAGAACGAATTG; human IL6 (antisense): CCA TCTTTGGAAGGTTCAGGTTG; human TNF-α (sense):

TTCTGCCTGCTGCACTTTGG; human TNF-α (antisense):

TGATGGCAGAGAGGAGGTTG; human OPN (sense): CCACA TGGCTAAACCCTGACC; human OPN (antisense):

CATGGCTTTCGTTGGACTTACTTG; human MMP12 (sense):

GGAATCCTAGCCCATGCTTTT; human MMP12 (antisense):

CATTACGGCCTTTGGATCACT; human TIMP2 (sense): TCCT CTTGATAGGGTTGCCA; human TIMP2 (antisense): CGTTTTGC AATGCAGATGTA; human S100A4 (sense): GATGAGCAACTTG GACAGCAA; human S100A4 (antisense):

CTGGGCTGCTTATCTGGGAAG; human CTGF (sense): CAGCA TGGACGTTCGTCTG; human CTGF (antisense): AACCACGGT TTGGTCCTTGG; human CDH1 (sense):

CGAGAGCTACACGTTCACGG; human CDH1 (antisense): GGG TGTCGAGGGAAAAATAGG; human GAPDH (sense): GTCGGT GTGAACGGATTTG; human GAPDH (antisense): TCCCATT CTCAGCCTTGAC. For *MALAT1/Malat1*, mouse and human Taqman assays were used (Thermo Fisher Scientific, Waltham, MA). Gene expression levels were normalized to *β-actin/GAPDH* and quantified using the delta delta Ct method.

#### GapmeRs

The LNA GapmeRs for *MALAT1* (human *MALAT1*: transcript variant ENST00000619449, mouse *Malat1*: transcript variant ESMUST00000173314) were obtained from Exiqon (Vedbaek, Denmark). Negative control A GapmeR was used as negative control.

#### Western blot

Western blot was performed on lysates harvested in lysis buffer (50 mM Tris-HCl pH7.5, 150 mM NaCl, 1% SDS, 0.5% deoxycholate, and 0.5% Triton X-100) with the addition of protease inhibitors (cOmplete protease inhibitor cocktail, Roche, Basel, Switzerland). BCA Protein Assay Kit (Pierce) was used to determine total protein content, and 5–20 μg. of total protein was applied on Any-kD Mini-PROTEAN TGX Precast SDS page gels (Bio-Rad). Gels were transferred to nitrocellulose membranes using the Trans-Blot Turbo Transfer System (Bio-Rad) and blocked with 5% non-fat milk powder in PBS with 0.01% of Tween (PBST). Primary antibodies against the following proteins were used: α-SMA (R&D, Minneapolis, MN, USA) and GAPDH (Cell Signaling Technology, Leiden, The Netherlands). Membranes were incubated with these primary antibodies overnight at 4°C, followed by incubation with appropriate secondary HRP-labeled antibodies for 1 h at room temperature. Upon PBST washing, membranes were incubated with SuperSignal West Dura Chemiluminescent Substrate (Thermo Fisher Scientific). Quantification of the protein bands was performed using ImageJ software and normalized to GAPDH.

#### RNA immunoprecipitation

Human SUZ12 was immunoprecipitated from HUVECs. All immunoprecipitations performed using the MagnaRIP RNA-Binding Protein Immunoprecipitation Kit (EMD Millipore, Burlington, MA) according to the manufacturers' instructions. Briefly, an antibody targeting human SUZ12 (Cell Signaling, ab12073) or an isotype-matched control antibody (Sigma, 12–370) were bound to magnetic beads and incubated with lysed cells at 4°C for 24 h. Beads were isolated and cleaved from the bound proteins by proteinase K, and coprecipitated RNA was purified. qPCR analysis of total RNA was performed to detect enrichment of *MALAT1* and control genes in

the protein-of-interest precipitated fraction, which was determined as percentage of 1% input control.

### Immunohistochemistry

Kidneys were fixed in 4% PFA for 1 h at 4°C, cryopreserved in 20% sucrose, and frozen in liquid nitrogen. Five micrometer sections were stained for α-SMA, MECA32, and F4/80 using a mouse fluorescein isothiocyanate-conjugated antibody against α-SMA (Sigma) or a specific antibody against MECA32 (Becton Dickinson, Franklin Lakes, New Jersey, USA) or F4/80 (Abcam, Cambridge, UK) followed by the corresponding Alexa or NovaRed-secondary antibodies (Invitrogen). For Picro Sirus Red (PSR), kidneys were sagittally sectioned and fixed in 10% neutral buffered formalin, processed, and embedded in paraffin wax. After sectioning, sections were dehydrated, xylene-cleared and mounted, and imaged using 3DHISTECH Pannoramic MIDI Scanner (Sysmex). PSR staining for collagen deposition was performed by 1 h incubation of the sections in 0.1% Sirius Red solution, followed by 2 rinses in 1% acidic acid. Stainings were quantified over the entire kidney section using HistoQuant software (3DHISTECH, Hungary) or ImageJ software (NIH, Bethesda, MD, USA). Staining of HUVECs for VE-cadherin and F-actin was performed by seeding HUVECs on an 8-well ibidi plate and fixation with 4% paraformaldehyde (Added Pharma, 1642810) and permeabilization with Triton X-100 (Merck, 11869). 5% BSA was used to block non-specific antigens. HUVECs were incubated with mouse anti-human CD144 (VE-cadherin) primary antibody (BD Pharmingen, 555661) and then secondary IgG goat anti-mouse Alexa 488 (Invitrogen, A32723), 1:200 Phalloidin-TRITC (Sigma, P1951), and 1:2,000 Hoechst (Thermo Fisher, H3569). Images were taken using a high-content confocal microscope (Molecular Devices, ImageXpress Micro Confocal) for determining FAJs: the cell boundary was identified by VE-cadherin staining, and the length of the boundary was measured using the length measurement tool in ImageJ. The cell-cell junction pattern is determined by the relative orientation of F-actin and membrane. The F-actin on FAJ was perpendicular to the cell membrane, while the F-actin bundle is parallel to the cell membrane on mature junctions.[21] The FAJ length of the selected cell was measured, and the ratio of the FAJ length to the total length was calculated to obtain the FAJ ratio of each cell.

### Vascular leakage and angiogenesis assay

HUVECs were used to culture 3D capillary-like vessels on the Organoplate microfluidic system (Mimetas, 9603-400B) and used to assess leakage/permeability based on the method described by van Duinen et al.[29] After 7 days, the capillary-like vessels were used to assess permeability by adding Alexa 555-labeled albumin (75 μg/mL, Invitrogen, A34786) to the perfusion channel followed by assessment of leakage of albumin to the gel channel over a time period of 30 min using the ImageXpress confocal microscope (Molecular Devices). Quantification of vascular leakage was performed using ImageJ as previously described.[29] For the angiogenesis assay, the same platform with the 3D capillary-like vessels on the Organoplate microfluidic system was used and performed as described previously.[54] In short, angiogenic sprouts were stimulated with VEGF + bFGF + S1P for 4 days, where the last 48 h were in the presence of *MALAT1* and control GapmeRs. Angiogenic factors were used in the following concentrations: 50 ng/mL for VEGF, 50 ng/mL for bFGF, and 500 nM for S1P. Sprouting was visualized with phalloidin staining (Sigma-Aldrich) and imaged using a high-content confocal microscope (Molecular Devices, ImageXpress Micro Confocal). Total sprouting area and average sprouting length were quantified using ImageJ by manually determining the distance between the microvessel and the (tip) cell sprouting furthest into the gel.

### Endothelial barrier function

Using the electric cell-substrate impedance sensing system (ECIS Zθ, Applied Biophysics) and ECIS plates (96W20idf PET, Applied Biophysics), endothelial barrier function was assessed by measuring trans-endothelial electrical resistance, as previously described.[53] Multiple frequency/time (MFT) mode was used to assess the barrier, and results are expressed as relative resistance at a frequency of 4,000 Hz. Using impedance data, the ECIS software was used for further mathematical modeling to calculate the cell morphological parameters of cell-cell (Rb) and cell-matrix (α) contacts.

### ELISA

The mouse Pro-Collagen I alpha 1 ELISA (Abcam) was performed on cell lysates according to the instructions of the manufacturer.

### Mitochondrial respiration

Oxygen consumption rate and corresponding analyses of basal respiration, maximal respiration, and proton leak were measured using the Seahorse XF96 analyzer and its associated Wave software (Agilent Technologies).

### ISH

Chromogenic *in situ* detection was performed on formalin-fixed paraffin-embedded mouse kidney tissue sections using the RNAscope ISH technology (Advanced Cell Diagnostics, Bio-Techne, Minneapolis, MN). 5-μm sections were used and deparaffinized followed by boiling with RNAscope Target Retrieval Reagent for 15 min at 99°C and subsequent protease digestion for 30 min at 40°C. Hybridization was performed at 40°C for 2 h with RNAscope Probe - Mm-Malat1 (313391, Advanced Cell Diagnostics). RNAscope Negative Control Probe_dapB (310043) and RNAscope Probe - Mm-Ppib (313911) were used as negative and positive controls, respectively. RNAscope 2.5 HD Reagent Kit (Brown) (322310) was used to visualize the bound probes.

### RNA-seq and pathway analysis

RNA-seq on FACS-sorted tdTomato-positive cells from mouse kidneys after the mice were treated with *MALAT1* or control GapmeR was performed by Novogene (Cambridge, UK). In short, sequencing libraries were generated using NEBNext Ultra RNA Library Prep Kit for Illumina (NEB, USA) following manufacturer's recommendations. mRNA was purified from total RNA using poly-T oligo-attached magnetic beads. Fragmentation was carried out using divalent cations under elevated temperature in NEBNext First Strand Synthesis Reaction Buffer (5×). First strand cDNA was synthesized

using random hexamer primer and M-MuLV Reverse Transcriptase (RNase H). Second strand cDNA synthesis was subsequently performed using DNA polymerase I and RNase H. Remaining overhangs were converted into blunt ends via exonuclease/polymerase activities. After adenylation of 3′ ends of DNA fragments, NEBNext adaptor with hairpin loop structure was ligated to prepare for hybridization. In order to select cDNA fragments of preferentially 150–200 bp in length, the library fragments were purified with AMPure XP system (Beckman Coulter, Beverly, USA). Then 3 μL USER Enzyme (NEB, USA) was used with size-selected, adaptor-ligated cDNA at 37°C for 15 min followed by 5 min at 95°C before PCR. Then PCR was performed with Phusion high-fidelity DNA polymerase, universal PCR primers, and index (X) primer. At last, PCR products were purified (AMPure XP system) and library quality was assessed on the Agilent Bioanalyzer 2100 system. Sequencing reads were aligned to the mouse genome (GRCm39 M33) using STAR (v.2.7.7a). Mapped reads were quantified for genomic features with featureCounts. For differential expression analysis, a quasi-likelihood negative binomial generalized log-linear model was applied using the edgeR package (v.3.18) in R (v.4.4.0). Read counts were normalized using the trimmed mean of M values method. Genes were considered differentially expressed if the contrast between conditions reached statistical significance, defined by a false discovery rate-adjusted *p* value of less than 0.05. All statistical analyses were performed in R. Normalized data were used for gene set and GO enrichment analysis. Additional (pathway) analyses, where indicated, were performed using Enrichr,[55] IPA software, and Rummagene.[24] Morpheus tool (Broad institute, https://software.broadinstitute.org/morpheus/) was used to visualize gene expression in a max projection heatmap.[56]

### Transcription factor motif enrichment, CatRAPID, and Hi-C

Discovery of enriched transcription factor-binding site motifs within lncRNA promoter regions (defined as 2,000 bp upstream of transcription start site) was performed by the analysis of motif enrichment tool,[57] using the JASPAR general database. The catRAPID algorithm[58] was used to determine RNA-binding domains and RNA-binding proteins in Malat1 and MALAT1. We visualized the 3-dimensional (3D) architecture within the genetic locus of MALAT1 and Malat1 using high-throughput chromosome conformation capture (Hi-C)[59] (data from the 3D genome browser[23]) in HUVECs[60] and C2C12.[61]

### ChIP

MEECs were used to perform ChIP on HMGA1 with 10 μg anti-HMGA1a/HMGA1b antibody-ChIP grade (ab4078, Abcam) or negative control IgG using the EZ-Magna ChIP A/G chromatin immunoprecipitation kit (Merck-Millipore) according to the instructions of the manufacturer. Malat1 DNA was determined using qPCR. Data were normalized to IgG-negative controls. PCR primers that were used to detect *Malat1* promoter region on the immunoprecipitated DNA were as follows: mmu-*Malat1*-promoter-bus/ree-fw AGCTTTAATCCAGCACTTGTGTAAG; mmu-*Malat1*-promoter-bus/ree-rev GGAGGTCCAGTGTTAGACCATT; mmu-*Malat1*-promoter-jaspar-fw GACGGGTTCCGCGGTC; mmu-*Malat1*-promoter-jaspar-rev CCAGGTCTATCTCATCGCTTCC; mmu-*Malat1*-promoter-manabe-fw GAAACATCTGAAAAACTTGGGGCT; mmu-*Malat1*-promoter-manabe-rev GGCCTCTTGGACCTTGCTAATA.

### Statistical analyses

Results are expressed as mean ± standard error of the mean (SEM), unless otherwise indicated. Statistical analyses were performed using student's t test or one-way ANOVA, and specific tests used have been indicated in the figure legends. $p < 0.05$ was considered statistically significant. For IPA, bias corrected $Z$ scores were determined with a $Z$ score higher than 2 or lower than −2 being considered statistically significant.

## DATA AVAILABILITY

The authors declare that the main data supporting the findings of this study are available within the article and its supplemental information files, extra data are available from the corresponding author upon request.

## ACKNOWLEDGMENTS

R.B. was supported by grants from the Dutch Kidney Foundation (14OIP13 and 20OK015) and EFSD/Novo Nordisk Foundation Future Leaders Awards Program (NNF23SA0087433). R.B. and A.J.v.Z. were further supported by a grant from the European Foundation for the Study of Diabetes (EFSD). C.v.S. is supported by a grant from the American Heart Association (23SCEFIA1153739).

## AUTHOR CONTRIBUTIONS

Conceptualization, R.B., A.J.v.Z., and C.v.S.; methodology, R.B., Q.Z., L.A.K.v.d.P., M. G., D.P., W.G.R., H.K., A.M.v.O.-R., A.L., J.A.d.K., R.C.S., and L.M.H.; investigation, R.B., Q.Z., W.S., A.K., J.M.G.J.D., J.A.d.K., and L.A.K.v.d.P.; writing – original draft, R.B. and Q.Z.; writing – review and editing, R.B., C.v.S., A.J.v.Z., and J.I.R.; funding acquisition, R.B. and A.J.v.Z.; supervision, R.B., A.J.v.Z., and C.v.S.

## DECLARATION OF INTERESTS

The authors declare no competing interests.

## SUPPLEMENTAL INFORMATION

Supplemental information can be found online at https://doi.org/10.1016/j.omtn.2025.102689.

## REFERENCES

1. GBD Chronic Kidney Disease Collaboration (2020). Global, regional, and national burden of chronic kidney disease, 1990-2017: a systematic analysis for the Global Burden of Disease Study 2017. Lancet *395*, 709–733.
2. Levey, A.S., and Coresh, J. (2012). Chronic kidney disease. Lancet *379*, 165–180.
3. Kalantar-Zadeh, K., Jafar, T.H., Nitsch, D., Neuen, B.L., and Perkovic, V. (2021). Chronic kidney disease. Lancet *398*, 786–802.
4. Long, D.A., Norman, J.T., and Fine, L.G. (2012). Restoring the renal microvasculature to treat chronic kidney disease. Nat. Rev. Nephrol. *8*, 244–250.
5. Choi, Y.J., Chakraborty, S., Nguyen, V., Nguyen, C., Kim, B.K., Shim, S.I., Suki, W. N., and Truong, L.D. (2000). Peritubular capillary loss is associated with chronic tubulointerstitial injury in human kidney: altered expression of vascular endothelial growth factor. Hum. Pathol. *31*, 1491–1497.
6. Seron, D., Alexopoulos, E., Raftery, M.J., Hartley, B., and Cameron, J.S. (1990). Number of interstitial capillary cross-sections assessed by monoclonal antibodies: relation to interstitial damage. Nephrol. Dial. Transplant. *5*, 889–893.
7. Rabelink, T.J., Wijewickrama, D.C., and de Koning, E.J. (2007). Peritubular endothelium: the Achilles heel of the kidney? Kidney Int. *72*, 926–930.

8. Ishii, Y., Sawada, T., Kubota, K., Fuchinoue, S., Teraoka, S., and Shimizu, A. (2005). Injury and progressive loss of peritubular capillaries in the development of chronic allograft nephropathy. Kidney Int. *67*, 321–332.

9. Lorenzen, J.M., and Thum, T. (2016). Long noncoding RNAs in kidney and cardiovascular diseases. Nat. Rev. Nephrol. *12*, 360–373.

10. Bink, D.I., Pauli, J., Maegdefessel, L., and Boon, R.A. (2023). Endothelial microRNAs and long noncoding RNAs in cardiovascular ageing. Atherosclerosis *374*, 99–106.

11. Mattick, J.S., Amaral, P.P., Carninci, P., Carpenter, S., Chang, H.Y., Chen, L.L., Chen, R., Dean, C., Dinger, M.E., Fitzgerald, K.A., et al. (2023). Long non-coding RNAs: definitions, functions, challenges and recommendations. Nat. Rev. Mol. Cell Biol. *24*, 430–447.

12. Wang, P., Luo, M.L., Song, E., Zhou, Z., Ma, T., Wang, J., Jia, N., Wang, G., Nie, S., Liu, Y., et al. (2018). Long noncoding RNA lnc-TSI inhibits renal fibrogenesis by negatively regulating the TGF-beta/Smad3 pathway. Sci. Transl. Med. *10*, eaat2039.

13. Bijkerk, R., Au, Y.W., Stam, W., Duijs, J.M.G.J., Koudijs, A., Lievers, E., Rabelink, T. J., and van Zonneveld, A.J. (2019). Long Non-coding RNAs Rian and Miat Mediate Myofibroblast Formation in Kidney Fibrosis. Front. Pharmacol. *10*, 215.

14. Stanicek, L., Lozano-Vidal, N., Bink, D.I., Hooglugt, A., Yao, W., Wittig, I., van Rijssel, J., van Buul, J.D., van Bergen, A., Klems, A., et al. (2020). Long non-coding RNA LASSIE regulates shear stress sensing and endothelial barrier function. Commun. Biol. *3*, 265.

15. Wang, Y., Nakayama, M., Pitulescu, M.E., Schmidt, T.S., Bochenek, M.L., Sakakibara, A., Adams, S., Davy, A., Deutsch, U., Lüthi, U., et al. (2010). Ephrin-B2 controls VEGF-induced angiogenesis and lymphangiogenesis. Nature *465*, 483–486.

16. Groeneweg, K.E., Au, Y.W., Duijs, J.M.G.J., Florijn, B.W., van Kooten, C., de Fijter, J. W., Reinders, M.E.J., van Zonneveld, A.J., and Bijkerk, R. (2020). Diabetic nephropathy alters circulating long noncoding RNA levels that normalize following simultaneous pancreas-kidney transplantation. Am. J. Transplant. *20*, 3451–3461.

17. Hopper, R.K., Moonen, J.R.A.J., Diebold, I., Cao, A., Rhodes, C.J., Tojais, N.F., Hennigs, J.K., Gu, M., Wang, L., and Rabinovitch, M. (2016). Pulmonary Arterial Hypertension, Reduced BMPR2 Promotes Endothelial-to-Mesenchymal Transition via HMGA1 and Its Target Slug. Circulation *133*, 1783–1794.

18. Qi, C., Bin, L., Yang, Y., Yang, Y., Li, J., Zhou, Q., Wen, Y., Zeng, C., Zheng, L., Zhang, Q., et al. (2016). Glipizide suppresses prostate cancer progression in the TRAMP model by inhibiting angiogenesis. Sci. Rep. *6*, 27819.

19. Camos, S., Gubern, C., Sobrado, M., Rodriguez, R., Romera, V.G., Moro, M.A., Lizasoain, I., Serena, J., Mallolas, J., and Castellanos, M. (2014). The high-mobility group I-Y transcription factor is involved in cerebral ischemia and modulates the expression of angiogenic proteins. Neuroscience *269*, 112–130.

20. Wilson, P.C., Wu, H., Kirita, Y., Uchimura, K., Ledru, N., Rennke, H.G., Welling, P. A., Waikar, S.S., and Humphreys, B.D. (2019). The single-cell transcriptomic landscape of early human diabetic nephropathy. Proc. Natl. Acad. Sci. USA *116*, 19619–19625.

21. Huveneers, S., Oldenburg, J., Spanjaard, E., van der Krogt, G., Grigoriev, I., Akhmanova, A., Rehmann, H., and de Rooij, J. (2012). Vinculin associates with endothelial VE-cadherin junctions to control force-dependent remodeling. J. Cell Biol. *196*, 641–652.

22. Zhang, B., Arun, G., Mao, Y.S., Lazar, Z., Hung, G., Bhattacharjee, G., Xiao, X., Booth, C.J., Wu, J., Zhang, C., and Spector, D.L. (2012). The lncRNA Malat1 is dispensable for mouse development but its transcription plays a cis-regulatory role in the adult. Cell Rep. *2*, 111–123.

23. Wang, Y., Song, F., Zhang, B., Zhang, L., Xu, J., Kuang, D., Li, D., Choudhary, M.N. K., Li, Y., Hu, M., et al. (2018). The 3D Genome Browser: a web-based browser for visualizing 3D genome organization and long-range chromatin interactions. Genome Biol. *19*, 151.

24. Clarke, D.J.B., Marino, G.B., Deng, E.Z., Xie, Z., Evangelista, J.E., Ma'ayan, A., and Rummagene, A. (2024). massive mining of gene sets from supporting materials of biomedical research publications. Commun. Biol. *7*, 482.

25. G Hendrickson, D., Kelley, D.R., Tenen, D., Bernstein, B., and Rinn, J.L. (2016). Widespread RNA binding by chromatin-associated proteins. Genome Biol. *17*, 28.

26. Fan, Y., Shen, B., Tan, M., Mu, X., Qin, Y., Zhang, F., and Liu, Y. (2014). TGF-beta-induced upregulation of malat1 promotes bladder cancer metastasis by associating with suz12. Clin. Cancer Res. *20*, 1531–1541.

27. West, J.A., Davis, C.P., Sunwoo, H., Simon, M.D., Sadreyev, R.I., Wang, P.I., Tolstorukov, M.Y., and Kingston, R.E. (2014). The long noncoding RNAs NEAT1 and MALAT1 bind active chromatin sites. Mol. Cell *55*, 791–802.

28. Bijkerk, R., de Bruin, R.G., van Solingen, C., Duijs, J.M.G.J., Kobayashi, K., van der Veer, E.P., ten Dijke, P., Rabelink, T.J., Goumans, M.J., and van Zonneveld, A.J. (2012). MicroRNA-155 functions as a negative regulator of RhoA signaling in TGF-beta-induced endothelial to mesenchymal transition. MicroRNA *1*, 2–10.

29. van Duinen, V., van den Heuvel, A., Trietsch, S.J., Lanz, H.L., van Gils, J.M., van Zonneveld, A.J., Vulto, P., and Hankemeier, T. (2017). 96 perfusable blood vessels to study vascular permeability in vitro. Sci. Rep. *7*, 18071.

30. Goligorsky, M.S. (2024). Permissive role of vascular endothelium in fibrosis: focus on the kidney. Am. J. Physiol. Cell Physiol. *326*, C712–C723.

31. Michalik, K.M., You, X., Manavski, Y., Doddaballapur, A., Zörnig, M., Braun, T., John, D., Ponomareva, Y., Chen, W., Uchida, S., et al. (2014). Long noncoding RNA MALAT1 regulates endothelial cell function and vessel growth. Circ. Res. *114*, 1389–1397.

32. Wang, C., Qu, Y., Suo, R., and Zhu, Y. (2019). Long non-coding RNA MALAT1 regulates angiogenesis following oxygen-glucose deprivation/reoxygenation. J. Cell Mol. Med. *23*, 2970–2983.

33. Liu, J.Y., Yao, J., Li, X.M., Song, Y.C., Wang, X.Q., Li, Y.J., Yan, B., and Jiang, Q. (2014). Pathogenic role of lncRNA-MALAT1 in endothelial cell dysfunction in diabetes mellitus. Cell Death Dis. *5*, e1506.

34. Liu, S.X., Zheng, F., Xie, K.L., Xie, M.R., Jiang, L.J., and Cai, Y. (2019). Exercise Reduces Insulin Resistance in Type 2 Diabetes Mellitus via Mediating the lncRNA MALAT1/MicroRNA-382-3p/Resistin Axis. Mol. Ther. Nucleic Acids *18*, 34–44.

35. Yao, R.W., Wang, Y., and Chen, L.L. (2019). Cellular functions of long noncoding RNAs. Nat. Cell Biol. *21*, 542–551.

36. Lino Cardenas, C.L., Kessinger, C.W., Cheng, Y., MacDonald, C., MacGillivray, T., Ghoshhajra, B., Huleihel, L., Nuri, S., Yeri, A.S., Jaffer, F.A., et al. (2018). An HDAC9-MALAT1-BRG1 complex mediates smooth muscle dysfunction in thoracic aortic aneurysm. Nat. Commun. *9*, 1009.

37. Yin, Y., Lu, J.Y., Zhang, X., Shao, W., Xu, Y., Li, P., Hong, Y., Cui, L., Shan, G., Tian, B., et al. (2020). U1 snRNP regulates chromatin retention of noncoding RNAs. Nature *580*, 147–150.

38. Aguilar, R., Rosenberg, M., Levy, V., and Lee, J.T. (2025). An evolving landscape of PRC2-RNA interactions in chromatin regulation. Nat. Rev. Mol. Cell Biol. *26*, 631–642.

39. Dunn-Davies, H., Dudnakova, T., Baker, A.H., and Mitić, T. (2024). Epigenetic control of vascular endothelial function revealed by multi-omics. Eur. Heart J. *45*, 642–644.

40. Dunn-Davies, H., Dudnakova, T., Nogara, A., Rodor, J., Thomas, A.C., Parish, E., Gautier, P., Meynert, A., Ulitsky, I., Madeddu, P., et al. (2024). Control of endothelial cell function and arteriogenesis by MEG3:EZH2 epigenetic regulation of integrin expression. Mol. Ther. Nucleic Acids. *35*, 102173.

41. Morini, M.F., Giampietro, C., Corada, M., Pisati, F., Lavarone, E., Cunha, S.I., Conze, L.L., O'Reilly, N., Joshi, D., Kjaer, S., et al. (2018). VE-Cadherin-Mediated Epigenetic Regulation of Endothelial Gene Expression. Circ. Res. *122*, 231–245.

42. Liu, P., Zhang, B., Chen, Z., He, Y., Du, Y., Liu, Y., and Chen, X. (2020). A-induced lncRNA MALAT1 aggravates renal fibrogenesis in obstructive nephropathy through the miR-145/FAK pathway. Aging *12*, 5280–5299.

43. Xia, W., Chen, X., Zhu, Z., Chen, H., Li, B., Wang, K., Huang, L., Liu, Z., and Chen, Z. (2023). Knockdown of lncRNA MALAT1 attenuates renal interstitial fibrosis through miR-124-3p/ITGB1 axis. Sci. Rep. *13*, 18076.

44. Tripathi, V., Ellis, J.D., Shen, Z., Song, D.Y., Pan, Q., Watt, A.T., Freier, S.M., Bennett, C.F., Sharma, A., Bubulya, P.A., et al. (2010). The nuclear-retained noncoding RNA MALAT1 regulates alternative splicing by modulating SR splicing factor phosphorylation. Mol. Cell *39*, 925–938.

45. Cui, H., Banerjee, S., Guo, S., Xie, N., Ge, J., Jiang, D., Zörnig, M., Thannickal, V.J., and Liu, G. (2019). Long noncoding RNA Malat1 regulates differential activation of macrophages and response to lung injury. JCI Insight *4*, e124522.

46. Hu, M., Wang, R., Li, X., Fan, M., Lin, J., Zhen, J., Chen, L., and Lv, Z. (2017). LncRNA MALAT1 is dysregulated in diabetic nephropathy and involved in high glucose-induced podocyte injury via its interplay with beta-catenin. J. Cell Mol. Med. *21*, 2732–2747.

47. Kolling, M., Genschel, C., Kaucsar, T., Hubner, A., Rong, S., Schmitt, R., Sorensen-Zender, I., Haddad, G., Kistler, A., Seeger, H., et al. (2018). Hypoxia-induced long non-coding RNA Malat1 is dispensable for renal ischemia/reperfusion-injury. Sci. Rep. *8*, 3438.

48. Eißmann, M., Gutschner, T., Hämmerle, M., Günther, S., Caudron-Herger, M., Groß, M., Schirmacher, P., Rippe, K., Braun, T., Zörnig, M., and Diederichs, S. (2012). Loss of the abundant nuclear non-coding RNA MALAT1 is compatible with life and development. RNA Biol. *9*, 1076–1087.

49. Fawzy, M.S., Abu AlSel, B.T., Al Ageeli, E., Al-Qahtani, S.A., Abdel-Daim, M.M., and Toraih, E.A. (2020). Long non-coding RNA MALAT1 and microRNA-499a expression profiles in diabetic ESRD patients undergoing dialysis: a preliminary cross-sectional analysis. Arch. Physiol. Biochem. *126*, 172–182.

50. Uil, M., Hau, C.M., Ahdi, M., Mills, J.D., Kers, J., Saleem, M.A., Florquin, S., Gerdes, V.E.A., Nieuwland, R., and Roelofs, J.J.T.H. (2021). Cellular origin and microRNA profiles of circulating extracellular vesicles in different stages of diabetic nephropathy. Clin. Kidney J. *14*, 358–365.

51. Rodrigues, K.F., Pietrani, N.T., Fernandes, A.P., Bosco, A.A., de Sousa, M.C.R., de Fátima Oliveira Silva, I., Silveira, J.N., Campos, F.M.F., and Gomes, K.B. (2018). Circulating microparticles levels are increased in patients with diabetic kidney disease: A case-control research. Clin. Chim. Acta *479*, 48–55.

52. Burger, D., Turner, M., Xiao, F., Munkonda, M.N., Akbari, S., and Burns, K.D. (2017). High glucose increases the formation and pro-oxidative activity of endothelial microparticles. Diabetologia *60*, 1791–1800.

53. Vreeken, D., Bruikman, C.S., Stam, W., Cox, S.M.L., Nagy, Z., Zhang, H., Postma, R. J., van Zonneveld, A.J., Hovingh, G.K., and van Gils, J.M. (2021). Downregulation of Endothelial Plexin A4 Under Inflammatory Conditions Impairs Vascular Integrity. Front. Cardiovasc. Med. *8*, 633609.

54. van Duinen, V., Stam, W., Borgdorff, V., Reijerkerk, A., Orlova, V., Vulto, P., Hankemeier, T., and van Zonneveld, A.J. (2019). Standardized and Scalable Assay to Study Perfused 3D Angiogenic Sprouting of iPSC-derived Endothelial Cells In Vitro. J. Vis. Exp. https://doi.org/10.3791/59678.

55. Xie, Z., Bailey, A., Kuleshov, M.V., Clarke, D.J.B., Evangelista, J.E., Jenkins, S.L., Lachmann, A., Wojciechowicz, M.L., Kropiwnicki, E., Jagodnik, K.M., et al. (2021). Gene Set Knowledge Discovery with Enrichr. Curr. Protoc. *1*, e90.

56. Morpheus. https://software.broadinstitute.org/morpheus.

57. McLeay, R.C., and Bailey, T.L. (2010). Motif Enrichment Analysis: a unified framework and an evaluation on ChIP data. BMC Bioinf. *11*, 165.

58. Armaos, A., Colantoni, A., Proietti, G., Rupert, J., and Tartaglia, G.G. (2021). catRAPID omics v2.0: going deeper and wider in the prediction of protein-RNA interactions. Nucleic Acids Res. *49*, W72–W79.

59. Lieberman-Aiden, E., van Berkum, N.L., Williams, L., Imakaev, M., Ragoczy, T., Telling, A., Amit, I., Lajoie, B.R., Sabo, P.J., Dorschner, M.O., et al. (2009). Comprehensive mapping of long-range interactions reveals folding principles of the human genome. Science *326*, 289–293.

60. Rao, S.S.P., Huntley, M.H., Durand, N.C., Stamenova, E.K., Bochkov, I.D., Robinson, J.T., Sanborn, A.L., Machol, I., Omer, A.D., Lander, E.S., and Aiden, E. L. (2014). A 3D map of the human genome at kilobase resolution reveals principles of chromatin looping. Cell *159*, 1665–1680.

61. Barutcu, A.R., Maass, P.G., Lewandowski, J.P., Weiner, C.L., and Rinn, J.L. (2018). A TAD boundary is preserved upon deletion of the CTCF-rich Firre locus. Nat. Commun. *9*, 1444.

**OMTN, Volume *36***

## Supplemental information

# Targeting long non-coding RNA *MALAT1* preserves endothelial cell integrity and protects against kidney fibrosis

**Qiao Zhao, Loïs A.K. van der Pluijm, Morgane Gourvest, Atefeh Lafzi, Daniel Peled, Whitney G. Rubin, Juliette A. de Klerk, Roderick C. Slieker, Leen M. 't Hart, Wendy Stam, Annemarie M. van Oeveren-Rietdijk, Jacques M.G.J. Duijs, Angela Koudijs, Joris I. Rotmans, Hilal Kazan, Anton Jan van Zonneveld, Coen van Solingen, and Roel Bijkerk**

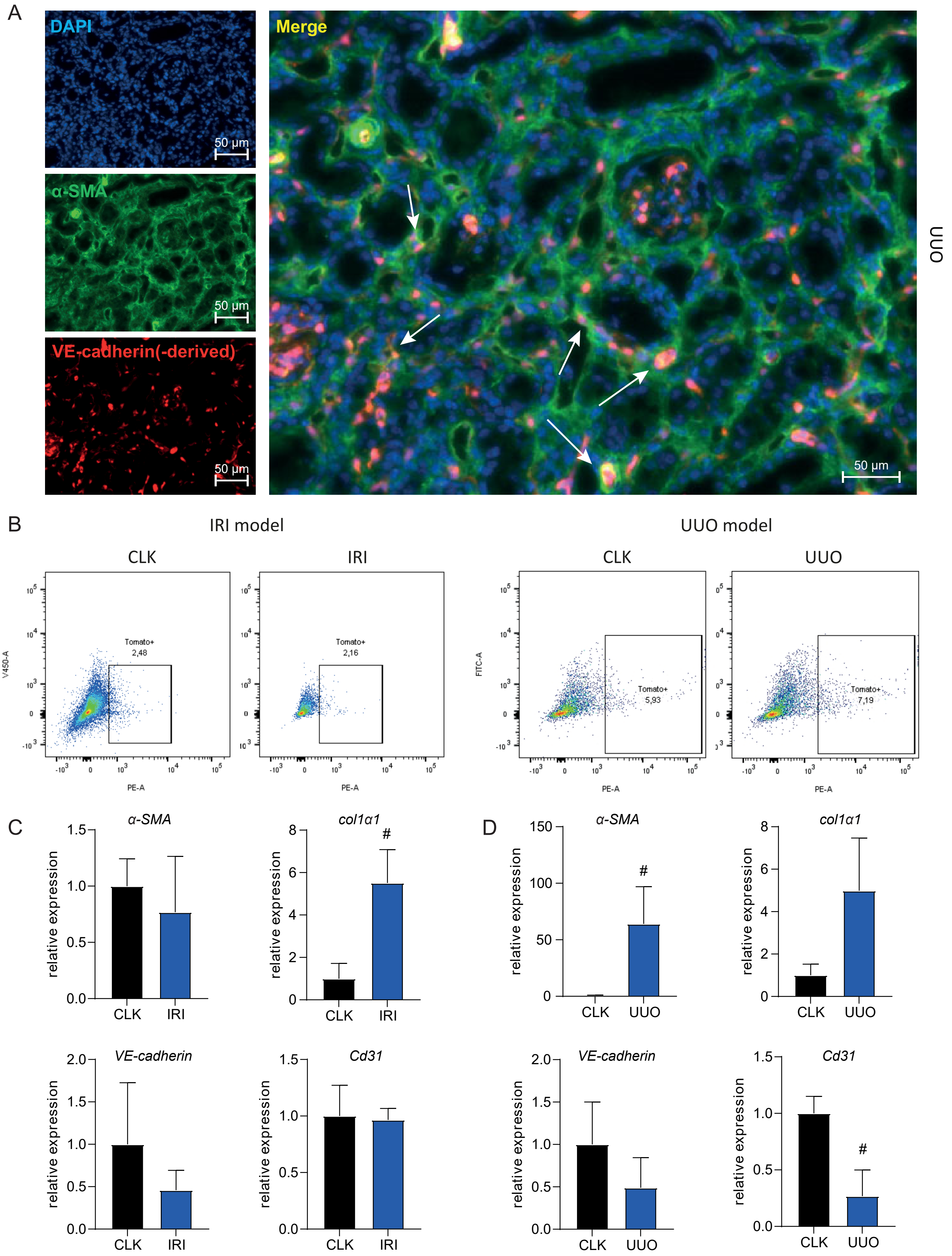


**Figure S1. Endothelial-to-mesenchymal transition in murine kidney fibrosis.** (A) representive image of co-localization (examples are indicated with arrows) of VE-cadherin-derived tomato positive cells and α-SMA stained positive cells in a UUO kidney. (B) Representative FACS plots for isolation of Tomato-positive VE-Cadherin-derived cells. (C-D) qPCR was performed for endothelial cell genes (*Cd31* and *Cdh5* (*VE-cadherin*)) and mesenchymal/ myofibroblast genes (*Acta2* (*α-SMA*) and *Col1α1*) on FACS sorted cells from VE-cadherin-tomato mice in the IRI model (C) and UUO model (D). #P<0.10.

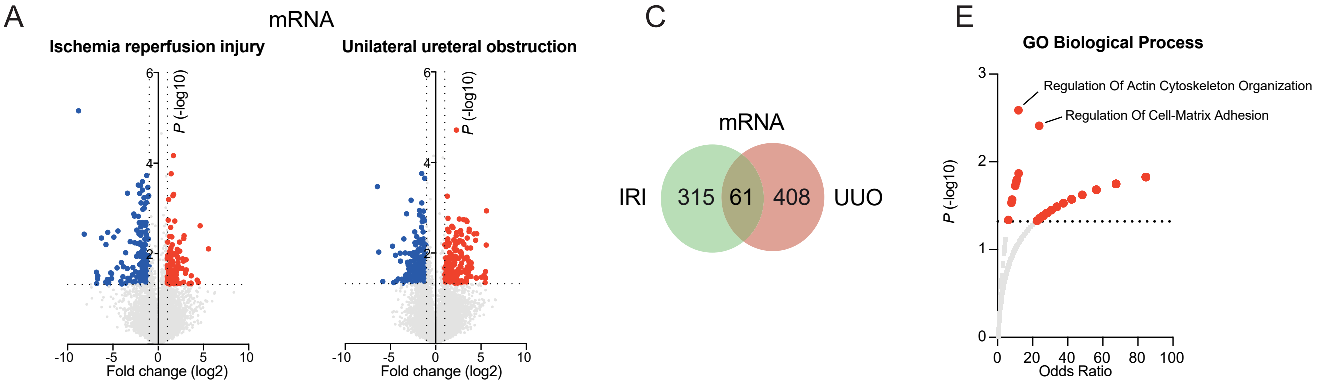


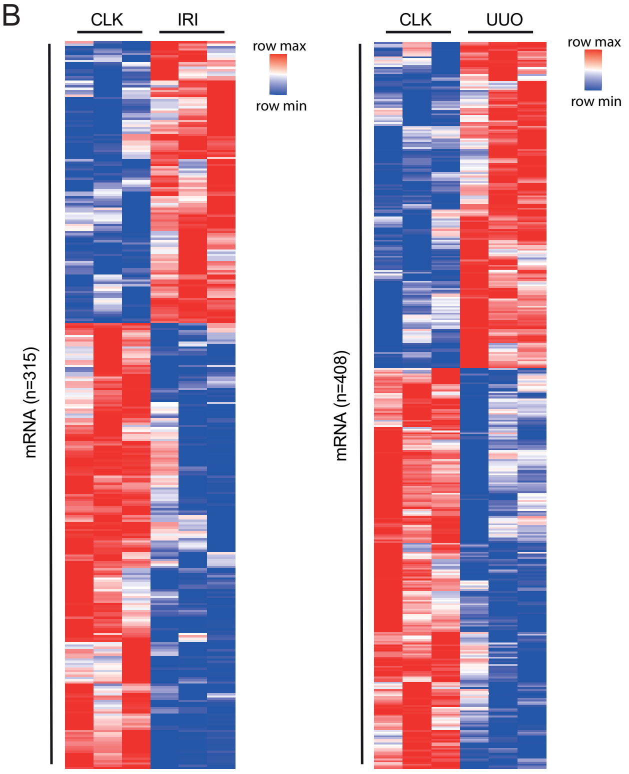


D

IRI

| Pathway | p-value |
|---|---|
| STAT3 Pathway | 1.30E-04 |
| S100 Family Signaling Pathway | 3.62E-04 |
| Molecular Mechanisms of Cancer | 4.03E-04 |
| WNK Renal Signaling Pathway | 5,60E-04 |
| Selenoamino acid metabolism | 7.37E-04 |

UUO

| Pathway | p-value |
|---|---|
| RhoA Signaling | 2.32E-05 |
| RHO GTPase cycle | 4.66E-05 |
| NCAM signaling for neurite out-growth | 3.00E-04 |
| S100 Family Signaling Pathway | 1.30E-03 |
| Triacylglycerol Degradation | 1.58E-03 |

**Figure S2. Differential mRNA expression in endothial (-derived) cells in IRI and UUO.** (A) Volcano Plots are visualizing differential mRNA expression between indicated conditions. The blue and red dots correspond to mRNAs with $P<0.05$ and $-1<\log FC>1$ that are down or up regulated, respectively. (B) Hierarchical clustering shows a distinguishable mRNA expression pattern in VE-cadherin derived cells in IRI and UUO compared to healthy contralateral kidneys (CLK) (n=3 per condition). (C) VENN diagram showing total number of differentially expressed genes per model as well as overlapping genes. (D) Ingenuity Pathway analyses indicates predicted enriched pathways. (E) GO analysis of biological pathways among the 61 overlapping genes between IRI and UUO are involved in actin cytoskeleton regulation and cell-matrix iadhesion.

A

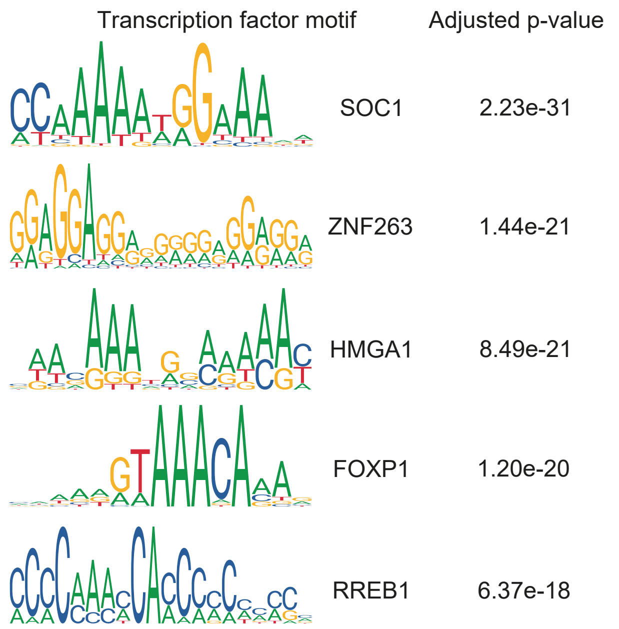


B

-821 ......TGGGAGAAAGAGAAAAACATCCTTT...... -796

Green - HMGA1 motif JASPAR

-1691 ......GAAAAATAAATAAGGAAGAAAGAAAA...... -1665

Red - HMGA1 motif (Reeves, 2000)

-249 ......TTGTGACCAGAAATTTTAAACCTATTT...... -222

Blue - HMGA1 motif (Manabe, 2009)

C

| Upstream Regulator | Predicted Activation State | Activation z-score |
|---|---|---|
| HMGA1 | Inhibited | -2.000 |
| LPS | Activated | 2.369 |
| OSM | Activated | 2.164 |
| CST5 | Activated | 2.000 |

D

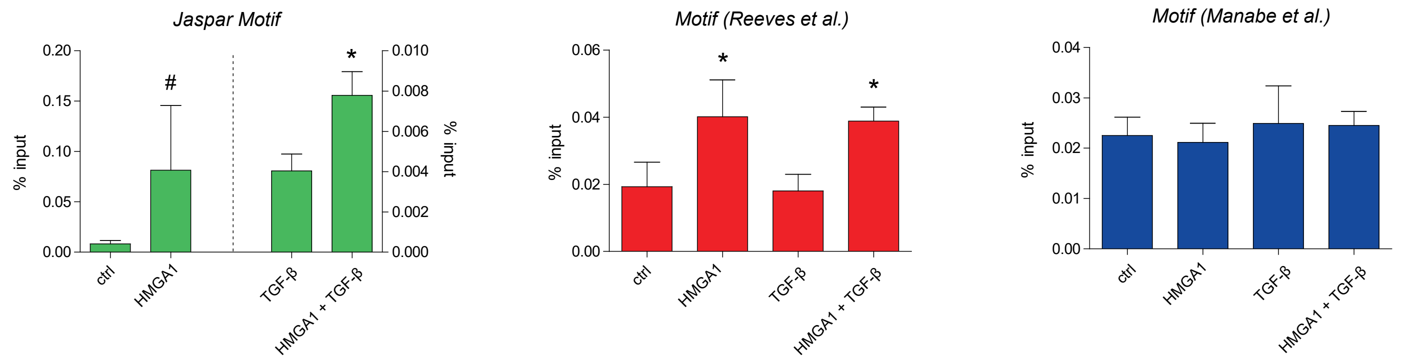


**Figure S3.** (A) Transcription factor motif enrichment analysis (JASPAR Core 2016) indicates enriched transcription factor motifs in promoter regions of differentially expressed lncRNAs. Top 5 (p-value) is depicted. (B) Within the promoter region of *Malat1* (defined as <2000 bp upstream of the start site) we found 3 potential HMGA1 binding motifs. In addition to the identified JASPAR motif for HMGA1, two additional potential binding motifs for HMGA1 were identfied from literature (Reeves, 2000, Environ Health Perspect & Manabe *et al.*, 2009, PLoS One). (C) Pathway analysis on differential mRNA profiles (Supplementary Figure 2) predicts HMGA1 to be a negatively regulated upstream regulator of differentially expressed genes in ECs in UUO. (D) PCR for *Malat1* promoter regions following HMGA1 ChIP, with IgG as negative control. *P<0.05, #P<0.10

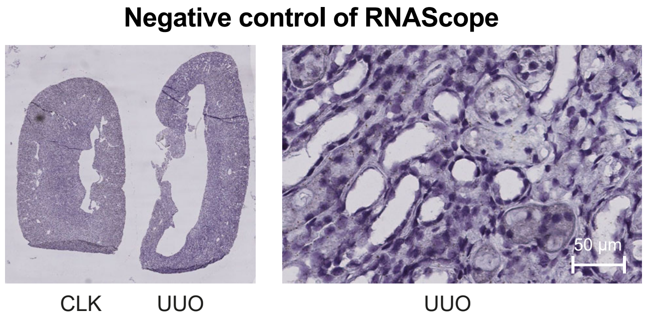


**Figure S4.** Negative control of RNAscope.

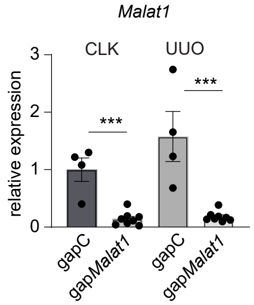


**Figure S5.** RT-qPCR analysis of *Malat1* in the kidney upon *Malat1* targeting GapmeR treatment (gap*Malat1*), compared to control GapmeR (gapC) treated mice. ***P<0.001. CLK = healthy contralateral kidney, UUO = fibrotic kidney from unilateral ureteral obstruction model. gapC = control GapmeR, gap*Malat1* = *Malat1* GapmeR.

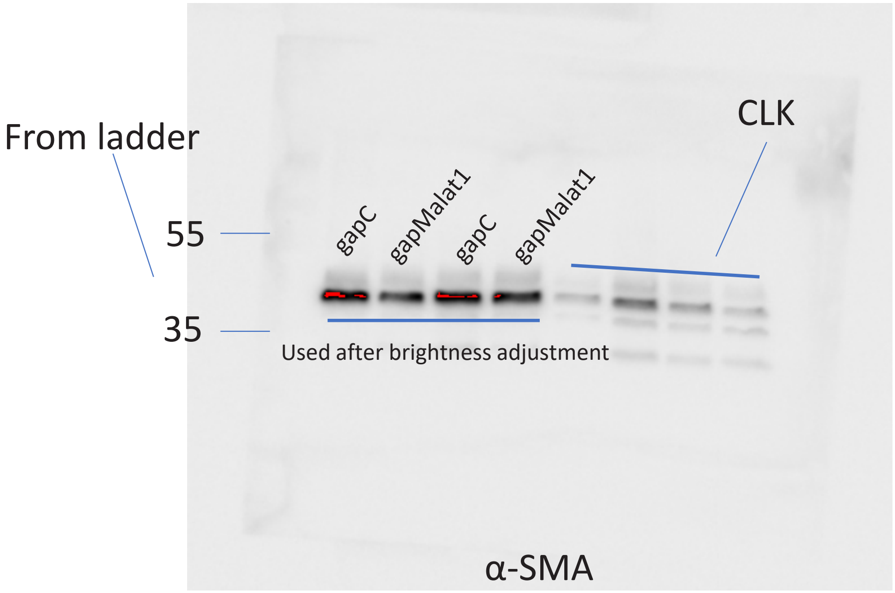
From ladder
CLK
55
35
gapC
gapMalat1
gapC
gapMalat1
Used after brightness adjustment
α-SMA

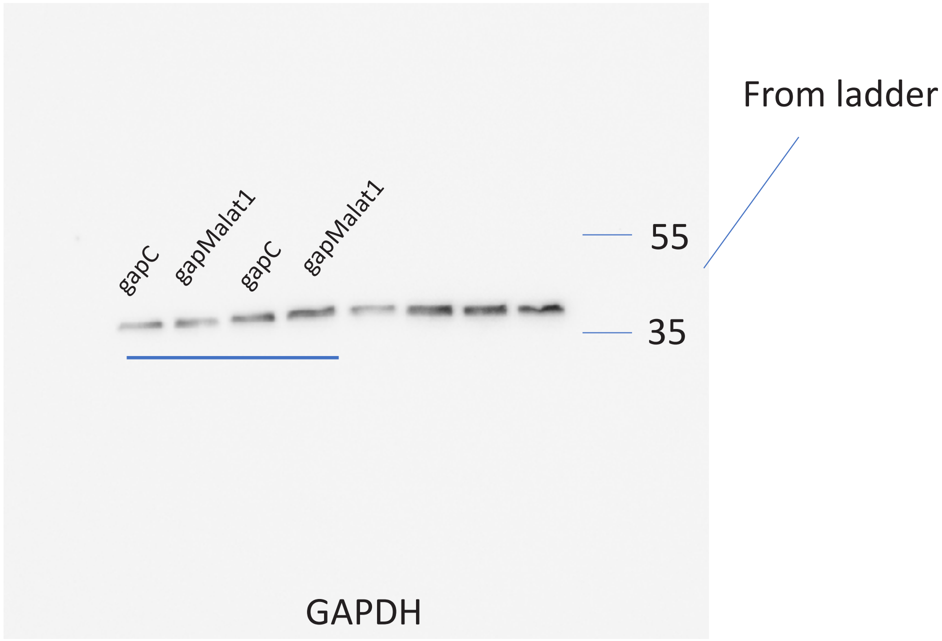
From ladder
gapC
gapMalat1
gapC
gapMalat1
55
35
GAPDH

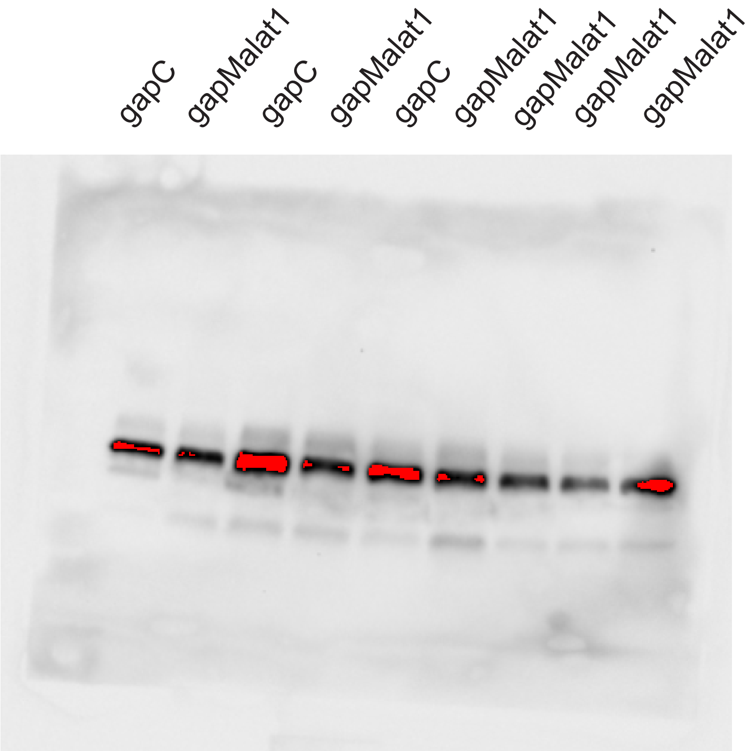


α-SMA

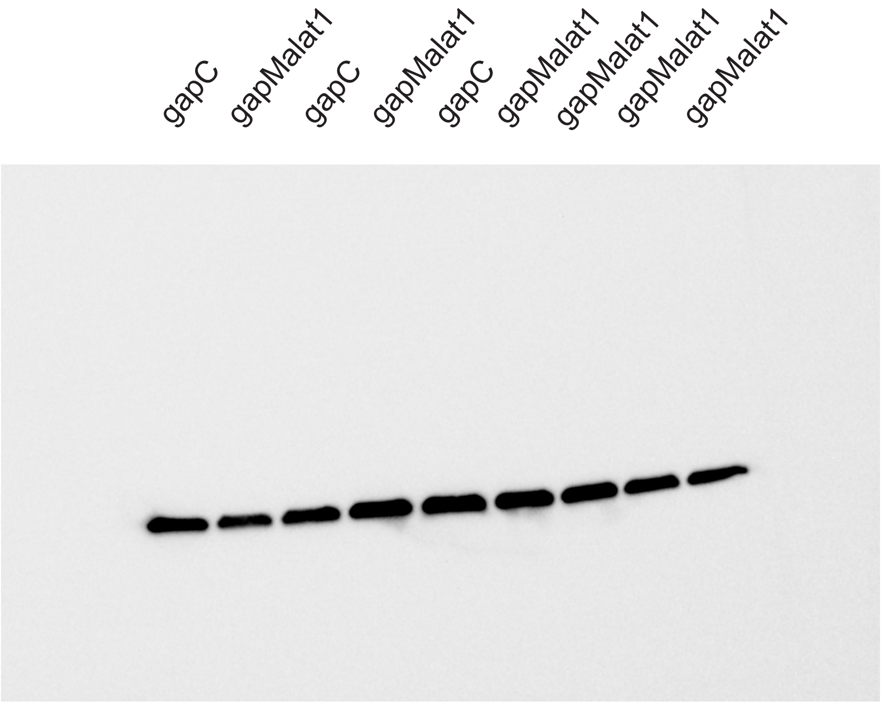


GAPDH

Additional samples

**Figure S6.** Full blots

Biorad chemidoc Image lab software; original blots for figure 4H

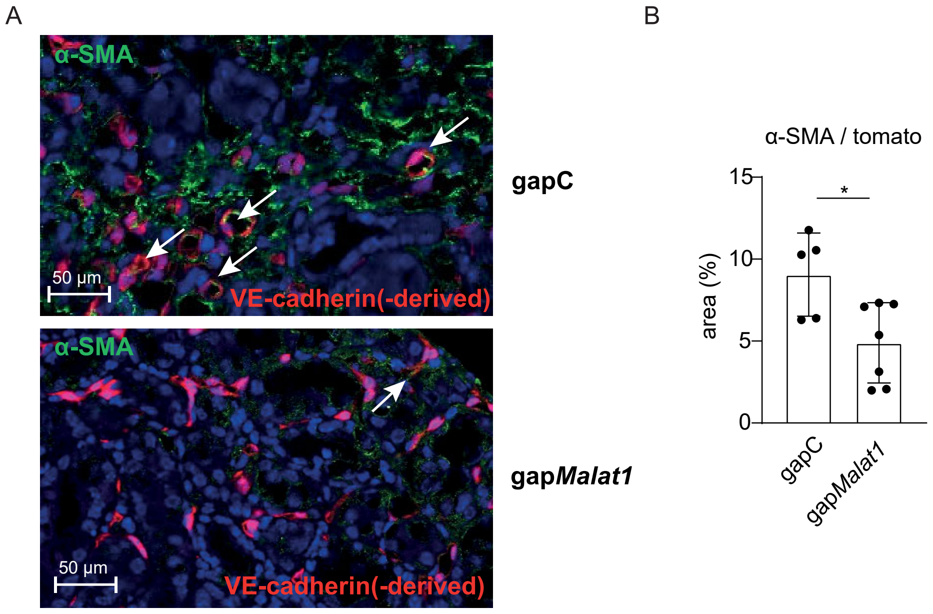


**Figure S7. *In vivo Malat1* knockdown reduces endothelial to mesenchymal transition.** (A-B) representive images (A) and quantification (B) of co-localization (examples are indicated with arrows) of VE-cadherin-derived tomato positive cells and α-SMA stained positive cells in a UUO kidneys from mice treated with control GapmeR (gapC) or GapmeR against *Malat1* (gap*Malat1*). *P<0.05.

| Ingenuity Canonical Pathways | Z-score |
|---|---|
| Oxidative Phosphorylation | -6.564 |
| Neutrophil Extracellular Trap Signaling Pathway | -4.621 |
| Mitochondrial Dysfunction | 4.608 |
| Phagosome Formation | 4.536 |
| CREB Signaling in Neurons | 4.158 |
| Granzyme A Signaling | 4.123 |
| EIF2 Signaling | -3.962 |
| Pathogen Induced Cytokine Storm Signaling Pathway | 3.772 |
| S100 Family Signaling Pathway | 3.592 |
| FAK Signaling | 3.528 |

**Figure S8. Ingenuity Pathway analysis.** Top 10 enriched canonical pathways as determined by ingenuity pathway analysis in differentially expressed genes in mouse kidney FACS-sorted ECs from mice treated with *Malat1*-targeting GapmeR**.**

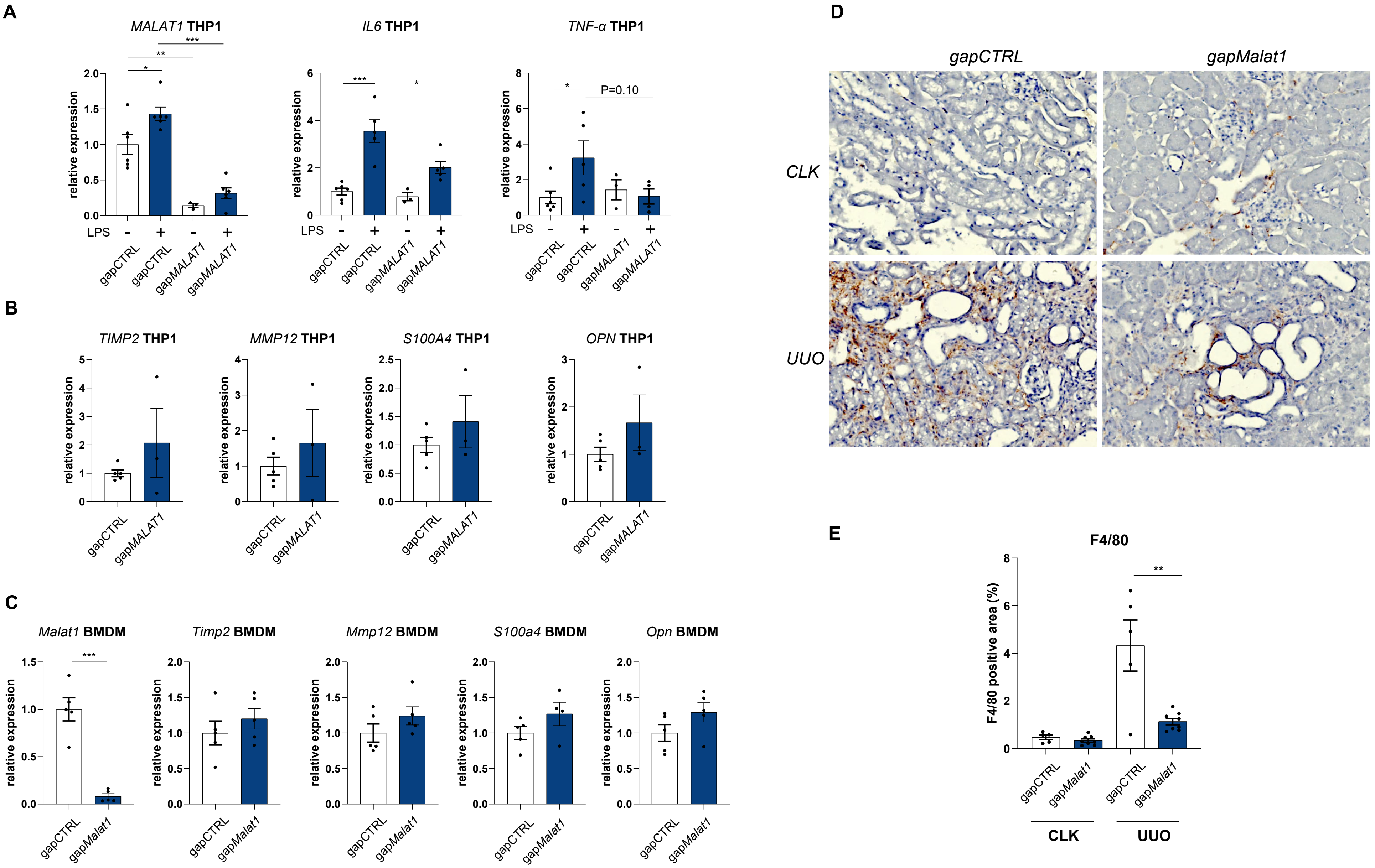


**Figure S9.** ***Malat1*** **knockdown decreases inflammatory phenotype of macrophages and results in less kidney infiltration of macrophages during injury** (A) *MALAT1* expression increases in PMA-differentiated, LPS-stimulated human THP1 cells and is decreased upon gapmer-mediated knockdown, which results in less LPS-induced *IL6* and *TNF-α* expression in THP1 cells. (B) Gene expression of profibrotic macrophage markers. (C) *Malat1* expression decreases upon gapmer-mediated knockdown in mouse bone-marrow derived macrophages (BMDM) and gene expressin of profibrotic macrophage markers in BMDM. (D) Representative microscopic images of kidney F4/80 macrophage staining, and corresponding quantification (E). *P<0.05, **P<0.01, ***P<0.001, CLK = (healthy) contralateral kidney, UUO = unilateral ureteral obstruction.

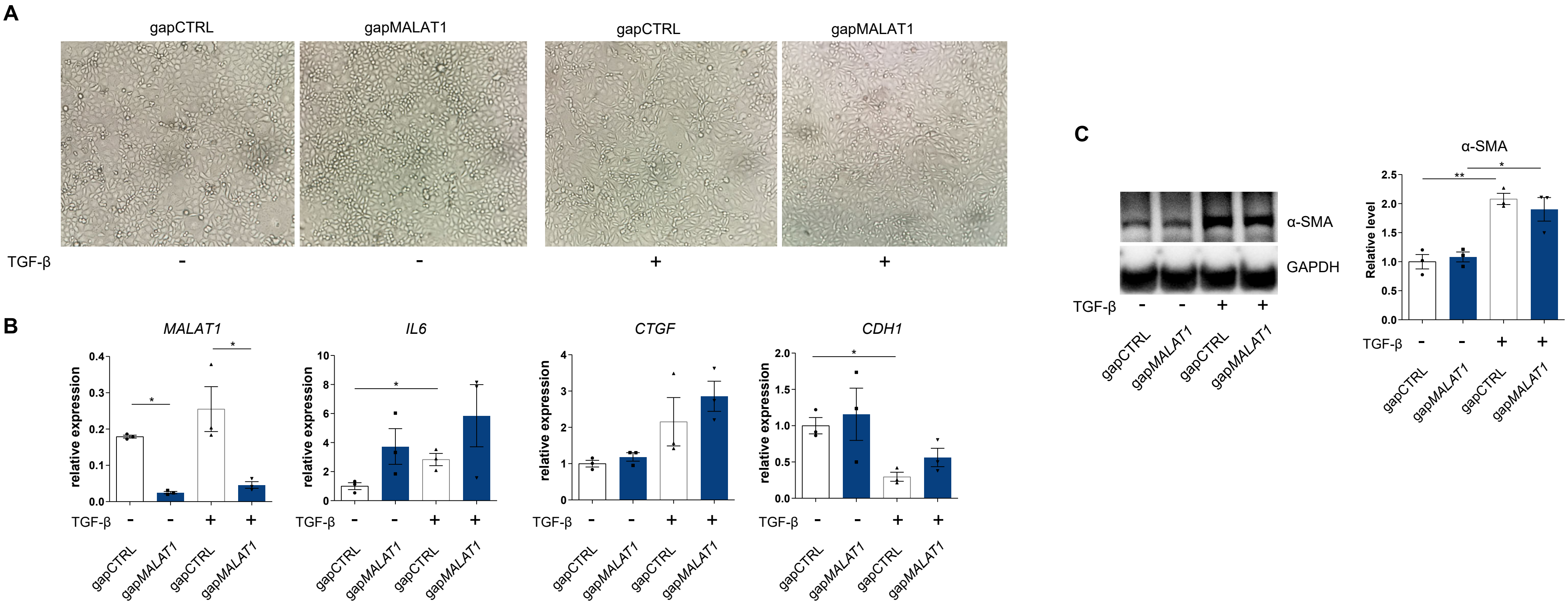


**Figure S10. *MALAT1* knockdown in HK2 cells.** (A) Representative microscopic images of HK2 epithelial cells with or without TGF-β stimulation and gapmer-mediated knockdown of MALAT1. (B) qRT-PCR plots of *MALAT1, IL6, CTGF and CDH1* expression. (C) Representative western blot for α-SMA and corresponding quantification. *P<0.05, **P<0.01.

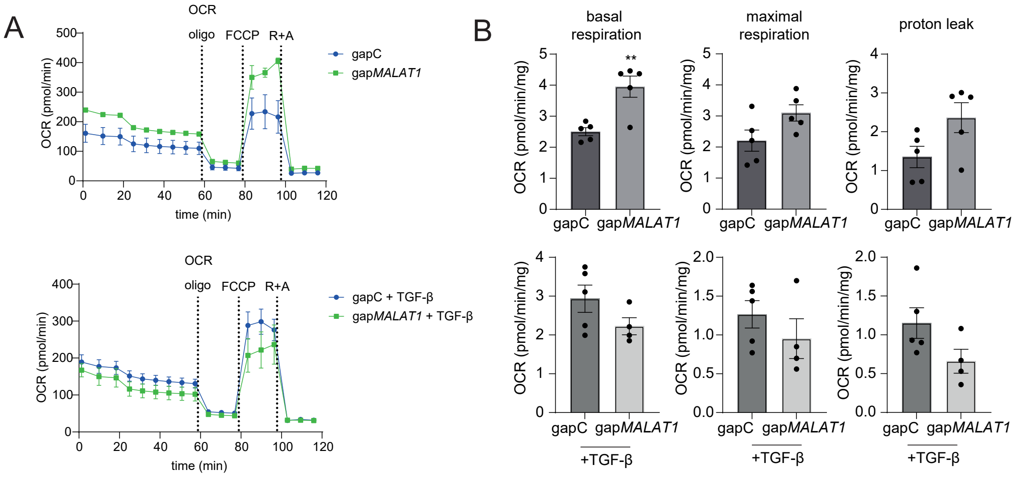


**Figure S11. Effect of *MALAT1* knockdown on mitochondrial function.** (A-B) Oxygen consumption rate, as determined by the Seahorse assay in HUVECs treated with GapmeR against *MALAT1* (gap*MALAT1*) or GapmeR control (gapC) with (lower panel) or without treatment with TGF-β (upper panel) (A) and corresponding quantification of basal respiration, maximal respiration and proton leak (B). **P<0.01

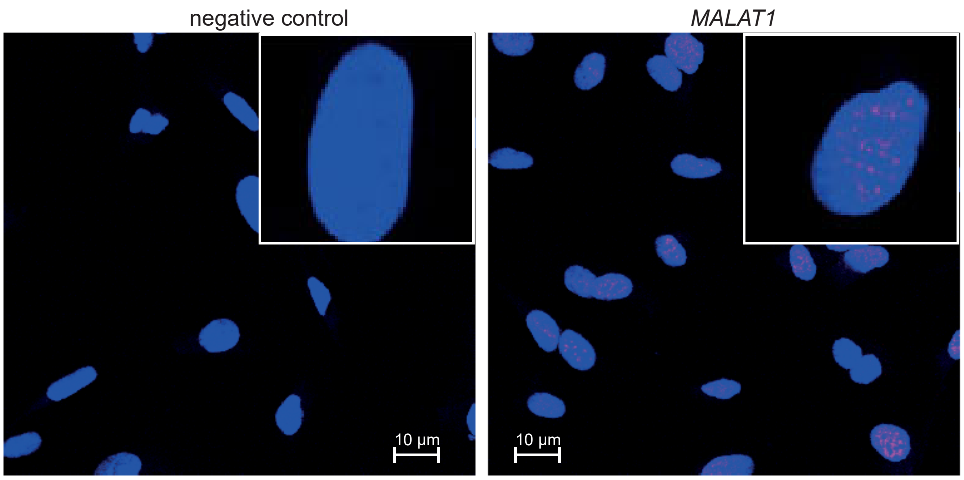


**Figure S12. FISH HUVEC.** Fluorescent in situ hybridization on HUVECs for *MALAT1* indicates nuclear localization. Upper right panel is zoomed in image of one nucleus.

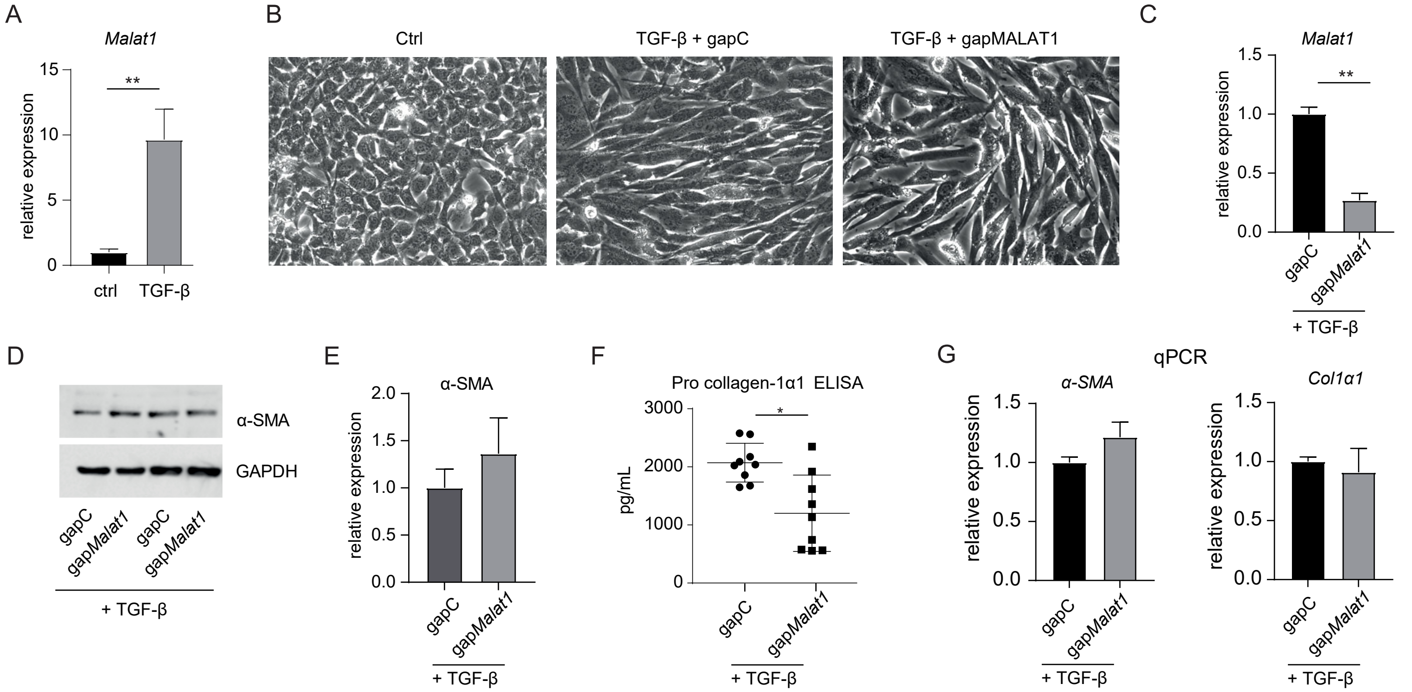


**Figure S13. *Malat1* knockdown during endothelial to mesenchymal transition does not alter α-SMA but decreases collagen deposition.** (A) *Malat1* expression increases in TGF-β stimulated mouse ECs. (B) Representative microscopic images of mouse ECs that undergo endothelial to mesenchymal transition upon TGF-β treatment, visible from the elongated morphology. (C) *Malat1* was inhibited using GapmeRs and compared to GapmeR control. (D-E) Representative western blots indicate TGF-β induces α-SMA protein expression, while this is not affected by *Malat1* inhibition. (F) *Malat1* knockdown decreased pro collagen1α1 levels as determined by ELISA. (G) Gene expression levels of myofibroblast marker *α-SMA* and fibrotic marker collagen1α1 did not change upon *Malat1* GapmeR. *P<0.05, **P<0.01.

Provided as Supplemental Excel Files:

**Table S1. Profiling lncRNAs IRI**

**Table S2. Profiling lncRNAs UUO**

**Table S3. Profiling mRNAs IRI**

**Table S4. Profiling mRNAs UUO**

**Table S5. RNA-seq EC gapM**
